# Network Pharmacology, Molecular Docking, and In Vitro Insights into the Potential of *Mitragyna speciosa* for Alzheimer’s Disease

**DOI:** 10.3390/ijms252313201

**Published:** 2024-12-08

**Authors:** Rahni Hossain, Kunwadee Noonong, Manit Nuinoon, Hideyuki J. Majima, Komgrit Eawsakul, Pradoldej Sompol, Md. Atiar Rahman, Jitbanjong Tangpong

**Affiliations:** 1 College of Graduate Studies, Walailak University, Nakhon Si Thammarat 80160, Thailand; rahni.ho@mail.wu.ac.th; 2 School of Allied Health Sciences, Walailak University, Nakhon Si Thammarat 80160, Thailand; kunwadee.no@wu.ac.th (K.N.); manit.nu@wu.ac.th (M.N.); hideyuki.ma@wu.ac.th (H.J.M.); 3 Research Excellence Center for Innovation and Health Product (RECIHP), Walailak University, Nakhon Si Thammarat 80160, Thailand; komgrit.ea@wu.ac.th; 4 Hematology and Transfusion Science Research Center, Walailak University, Nakhon Si Thammarat 80160, Thailand; 5 School of Medicine, Walailak University, Nakhon Si Thammarat 80160, Thailand; 6 Department of Pharmacology & Nutritional Sciences, College of Medicine, University of Kentucky, Lexington, KY 40536, USA; pradoldej.sompol@uky.edu; 7 Department of Biochemistry and Molecular Biology, University of Chittagong, Chittagong 4331, Bangladesh; atiar@cu.ac.bd

**Keywords:** Alzheimer’s disease, *Mitragyna speciosa*, network pharmacology, molecular docking, antioxidant, hydrogen peroxide, oxidative stress, SH-SY5Y cell

## Abstract

*Mitragyna speciosa* Korth. Havil (MS) has a traditional use in relieving pain, managing hypertension, treating cough, and diarrhea, and as a morphine substitute in addiction recovery. Its potential in addressing Alzheimer’s disease (AD), a neurodegenerative condition with no effective treatments, is under investigation. This study aims to explore MS mechanisms in treating AD through network pharmacology, molecular docking, and in vitro studies. Using network pharmacology, we identified 19 MS components that may affect 60 AD-related targets. The compound–target network highlighted significant interactions among 60 nodes and 470 edges, with an average node degree of 15.7. The KEGG enrichment analysis revealed Alzheimer’s disease (hsa05010) as a relevant pathway. We connected 20 targets to tau and β-amyloid proteins through gene expression data from the AlzData database. Docking studies demonstrated high binding affinities of MS compounds like acetylursolic acid, beta-sitosterol, isomitraphylline, and speciophylline to AD-related proteins, such as AKT1, GSK3B, NFκB1, and BACE1. In vitro studies showed that ethanolic (EE), distilled water (DWE), and pressurized hot water (PHWE) extracts of MS-treated 100 μM H_2_O_2_-induced SH-SY5Y cells significantly reduced oxidative damage. This research underscores the multi-component, multi-target, and multi-pathway effects of MS on AD, providing insights for future research and potential clinical applications.

## 1. Introduction

Alzheimer’s disease (AD) is a progressive neurodegenerative condition characterized by memory impairment, cognitive decline, and changes in behavior, which severely affect daily functioning [1]. In 2024, it is projected that approximately 6.9 million Americans aged 65 and older are living with AD, with estimates indicating that there could be 153 million cases globally by 2050 [2]. The development of AD is complex, involving a mix of genetic, environmental, and lifestyle contributors [3]. Critical theories include the cholinergic hypothesis, which focuses on neurotransmitter deficits, the accumulation of amyloid-beta (Aβ) and tau protein tangles, and oxidative stress in neuronal damage [4,5]. Reactive oxygen species (ROS) and reactive nitrogen species (RNS) are crucial signaling molecules in numerous physiological activities, produced by standard metabolic processes, such as oxidative phosphorylation, which is the primary source of cellular superoxide anion (O^2−^), hydroxyl radicals (•OH), and hydrogen peroxide (H_2_O_2_) [6,7]. Maintaining a proper balance of ROS/RNS is crucial for regulating intercellular communication and the immune response to invading microorganisms [8]. However, when ROS/RNS levels are excessive, they initiate a series of reactions that lead to the oxidation of lipids, proteins, DNA, and other vital molecules. This can result in oxidative stress, which is linked to neurodegenerative diseases, atherosclerosis, accelerated aging, and cancer [9,10]. Among various ROS/RNS, H_2_O_2_ is known to promote apoptosis in different cells, acts as a precursor to ROS that can easily move in and out of cells and tissues, can lead to hyperphosphorylation of tau protein, and induce the creation of harmful neurofibrillary tangles in brain cells [11,12]. Thus, the overproduction of free radicals compromises both the internal antioxidant defense mechanisms and necessitates external antioxidants from dietary sources, such as vitamins C and E and polyphenols, to prevent oxidative harm by acting as “free radical scavengers” to avert and repair damage caused by ROS and RNS [13]. Natural resources rich in phenolic compounds and pigments enhance antioxidant activity [14]. Currently, eight medications are available for AD, with cholinesterase inhibitors like Donepezil and NMDA receptor antagonists, such as Memantine, being the primary treatment options [15]. Although these can alleviate symptoms, they do not alter the course of the disease [2]. There is an urgent need for treatments that tackle the underlying causes of Alzheimer’s for lasting benefits. Bioactive compounds derived from plants, including phenols, glycosides, vitamins, and alkaloids, have shown significant promise in preventing plaque formation and enhancing cholinergic transmission [16].

*Mitragyna speciosa* (Korth.) (MS) Havil., commonly referred to as ‘ketom’ in Malaysia and ‘kratom’ in Thailand, is an herbaceous species in the coffee family (Rubiaceae) found throughout Southeast Asia [17,18]. MS, rich in bioactive compounds, has demonstrated various therapeutic effects and potential in managing multiple health issues [19]. Traditionally utilized in folk medicine for pain relief, it exhibits a range of pharmacological properties, including antioxidant, anti-inflammatory, analgesic, antidepressant, antimicrobial, antipsychotic, and antinociceptive effects with its various other bioactive components, such as oxindole alkaloids, saponins, flavonoids, phenolic compounds, tannins, and sterols [20,21]. The younger leaves of MS contain a greater concentration of active alkaloids than older ones, highlighting their potential as therapeutic agents [22].

**Figure 1:**
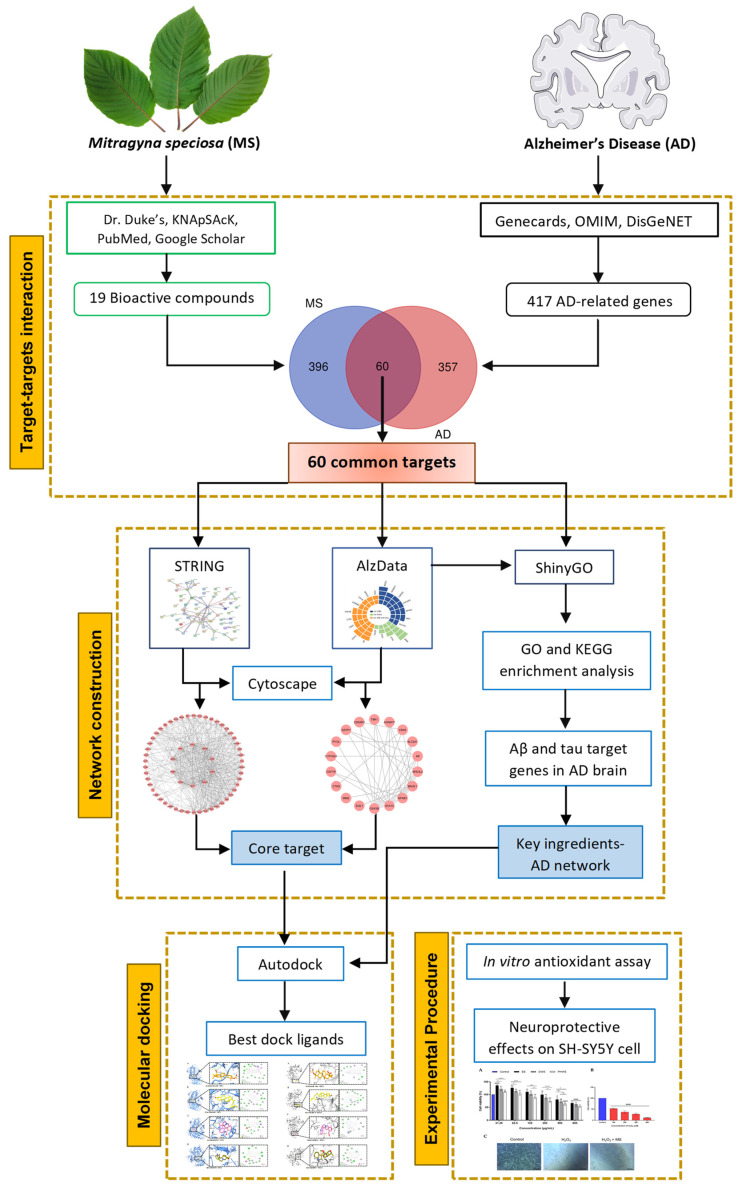
A block diagram represents the workflow of combining network pharmacology and molecular docking, followed by experimental validation to assess the efficacy of *M. speciosa* bioactive compounds in Alzheimer’s disease.

SH-SY5Y cells, derived from human neurons, replicate the human neuronal response to excess ROS and are utilized to investigate neurodegenerative diseases [23]. Continuous growth, immature neuronal proteins, and a low density of neuronal markers mark this cell line [24]. Network pharmacology, as first introduced by Hopkins, merges systems and computational biology to elucidate the connections between metabolites, targets, and diseases, facilitating drug discovery and providing insights into drug-organism interactions [25]. Integrating bioinformatics and network pharmacology through computational predictions is a robust approach to uncovering the relationships between biological mechanisms, complex diseases, and drug effects [26]. Molecular docking technology further investigates interactions between small molecules and larger biological macromolecules [26]. This study uniquely combines network pharmacology with molecular docking and in vitro validation to explore how *M. speciosa* may treat AD, focusing on identifying essential compounds and targets for effective multi-target strategies. The technological roadmap for this methodology is depicted in Figure 1. The findings from this research could offer additional understanding of the molecular processes and treatment strategies for AD and its associated effects.

## 2. Results

### 2.1. Screened M. speciosa Compounds and Predicted Targets for AD

*M. speciosa* generally contains a wide range of chemical compounds, with more than 100 variations observed in different geographical locations [18]. After thoroughly researching literature and databases, we identified 19 main phytochemicals in MS leaves. These compounds, which adhere to Lipinski’s Rule of Five (RO5) criteria, underwent toxicity screening using the Protox II webserver (Table 1). The toxicity screening rigorously targeted various endpoints, such as hepatotoxicity, carcinogenicity, immunotoxicity, mutation, and cytotoxicity. As per the globally harmonized system (GHS) of chemical classification, the compounds predicted acute oral toxicity classes were predominantly categorized as IV and V, clearly indicating safety and suitability from a drug perspective [27]. Additionally, we used the UniProt database to identify the genes corresponding to the target proteins. This resulted in retrieving 2121 predicted targets (Table S1) from Super-pred and Swiss Target Prediction databases for the 19 MS compounds. After combining and filtering the data, we obtained 456 distinct genes from two databases.

**Figure 2:**
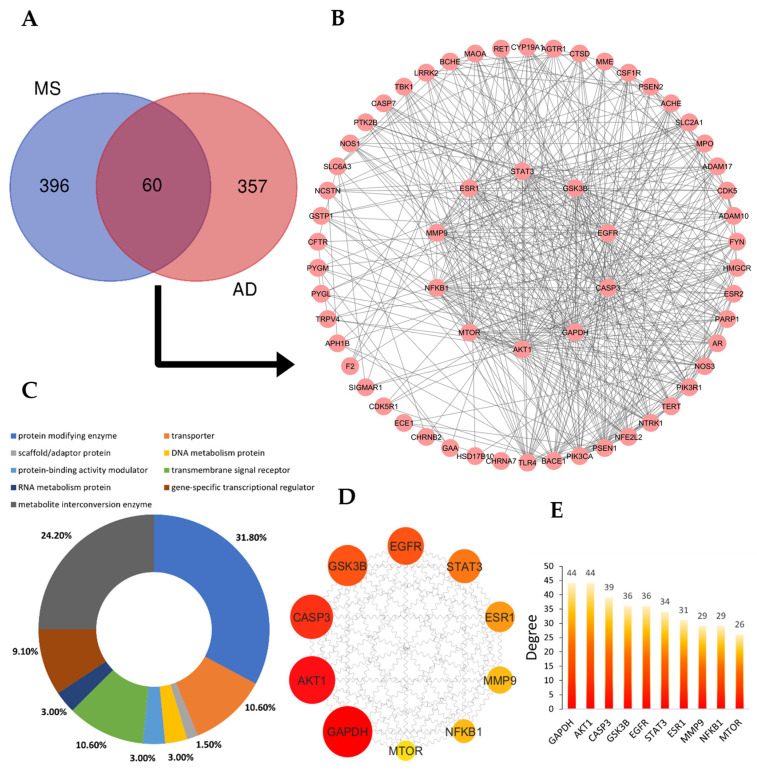
Protein–protein interaction network construction. (**A**) A Venn diagram highlighting the overlap between MS active components and AD-related targets. (**B**) A protein interaction network diagram of active components and disease targets (60) of MS was constructed. (**C**) Functional classification of AD genes and the active components of MS. (**D**) Top 10 hub genes ranked based on degree. (**E**) A plot representing the degree of each hub gene.

To find targets related to AD, we gathered various targets from the GeneCards database, DisGeNET, and OMIM database for AD. The genes associated with AD were obtained after eliminating duplicates, and we chose proteins that appeared in all databases. In total, 417 potential target proteins were anticipated (Table S2). Potential targets of MS active ingredients and AD were entered into Venny2.1 and presented in a Venn diagram (Figure 2A), revealing the shared targets of MS and AD. By merging common targets associated with MS and AD, we identified 60 common targets at the intersection of potential targets (Table 2 and Figure 2B).

### 2.2. Construction and Analysis of the Target Protein–Protein Interactions (PPI) Network

We have generated a network diagram illustrating the overlap of 60 genes associated with MS and AD targets (Figure 2B). These genes have been categorized based on cell function, resulting in nine distinct categories. Notably, protein-modifying enzymes, accounting for 31.8% of the identified genes, constitute the largest proportion (Figure 2C). This category encompasses critical enzymes such as GSK3B, TBK1, CASP7, PTK2B, F2, APH1B, PSEN1, AKT1, ADAM17, MTOR, BACE1, PSEN2, CDK5, FYN, ADAM10, CTSD, CASP3, and metalloproteases MME, ECE1, and MMP9. Human PPI data were obtained from STRING (Table S3) and filtered using medium confidence 0.4 to establish the PPI. The resulting PPI network comprises 60 nodes and 470 edges, representing distinct PPIs. The average node degree is determined to be 15.7, and the local clustering coefficient is 0.635, signifying the interconnectedness of the targets within the network. The PPI network was further analyzed using Cytoscape 3.10.2, and core targets were identified by CentiScaPe 2.2 Menu based on their degree, closeness, and betweenness centrality measures (Table S4). From this analysis, 10 hub genes were identified, including GAPDH, AKT1, CASP3, GSK3B, EGFR, STAT3, ESR1, MMP9, NFκB1, and MTOR, as depicted in Figure 2D,E.

### 2.3. Gene Ontology (GO) Biological Process and Kyoto Encyclopedia of Genes and Genomes (KEGG) Pathway Enrichment Analyses

**Figure 3:**
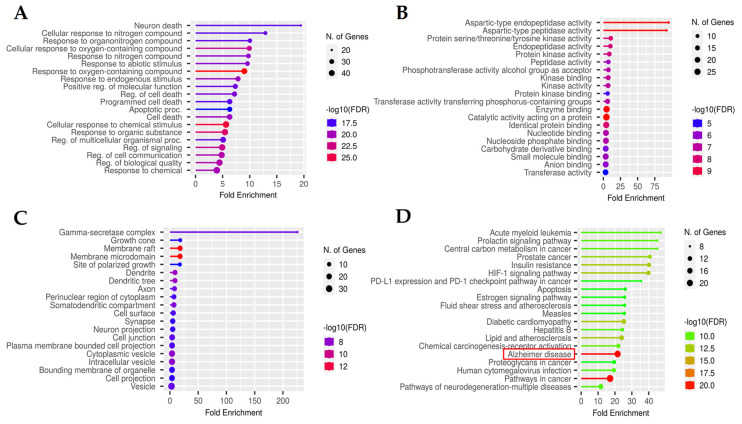
The enrichment analysis chart of the top 20 Gene Ontology (GO) categories for (**A**) biological processes (BP), (**B**) molecular functions (MF), (**C**) cellular component (CC), and (**D**) Kyoto Encyclopedia of Genes and Genomes (KEGG) of MS.

Functional enrichment analysis was conducted using ShinyGO 0.80 settings, applying three criteria and constraining the threshold of *p* ≤ 0.05, following the false discovery rate determined via the Benjamani–Hochberg method. The analysis yielded a total of 1061 items, including 872 items, including biological processes (BP) items, 88 molecular functions (MF) items, and 101 cellular components (CC) items. Our focus was on the top 20 GO BP (Figure 3A), GO MF (Figure 3B), and GO CC (Figure 3C) processes, with particular attention given to the significant KEGG pathways (Figure 3D). According to our study results of BP, the function of MS in AD mainly focused on the response to oxygen-containing compounds, cellular response to chemical stimulus, response to organic substances, cellular response to oxygen-containing compound, response to chemical, regulation of biological quality, regulation of signaling, response to nitrogen compound, regulation of cell communication, regulation of multicellular organismal process, regulation of cell death, etc. The MF items primarily included catalytic activity, enzyme binding, aspartic-type endopeptidase activity, protein binding, identical protein binding, endopeptidase activity, protein kinase activity, anion binding, kinase binding, nucleotide binding, etc. The confirmed KEGG pathways (Table S5) included Alzheimer’s disease (hsa05010), pathways in cancer (hsa05200), HIF-1 signaling pathway (hsa04066), insulin resistance (hsa04931), diabetic cardiomyopathy (hsa05415), lipid and atherosclerosis (hsa05417), prostate cancer (hsa05215), chemical carcinogenesis-receptor activation (hsa05207), acute myeloid leukemia (hsa05221), and pathways of neurodegeneration-multiple diseases (hsa05022), etc. Comprehensive KEGG analyses were also carried out to elucidate the involved signaling pathways, demonstrating significant connections with AD and neuroactive ligand-receptor interactions (Figure S1A) [28]. In Figure S1B, the network diagram from Cytoscape illustrates the compound-target pathway disease interactions of the effects of MS on AD. This intricate network comprises 381 nodes, including 19 compounds, 60 targets, and 19 pathways connected by 1668 edges. Additionally, Table S6 presents the hub genes of MS along with compounds and their associated key signaling pathways.

### 2.4. Bioinformatics Analysis of Target Proteins That Correlated with Aβ and Tau Pathology

**Figure 4:**
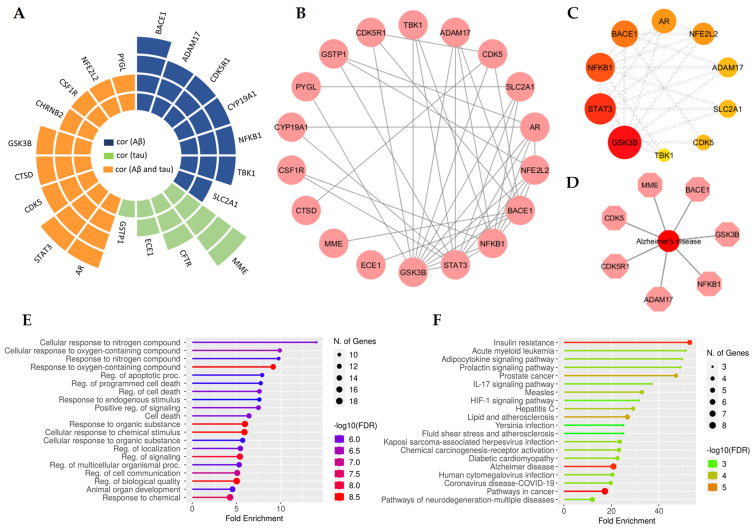
Targets associated with Aβ or tau were retrieved from the Alzdata database. (**A**) Radial bar plots of tau- or Aβ-related targets. (**B**) Diagram of the PPI network with tau or Aβ associated targets. (**C**) The top 10 hub genes. (**D**) Diagram of the core target PPI network associated with AD. (**E**) BP enrichment analysis. (**F**) KEGG enrichment analysis.

To narrow down the genes associated with the pathogenesis of AD, we sought to correlate the genes with the amyloid deposition hypothesis and tau phosphorylation hypothesis, as these are the primary hallmarks of AD [29]. We utilized the AlzData database to analyze the Aβ or tau pathology and identified 20 targets (Figure 4A–D). These 20 targets were further used to construct a PPI network and perform BP and KEGG analysis. The enrichment analysis of BP pathways included response to organic substances, regulation of biological quality, cellular response to chemical stimulus, response to oxygen-containing compounds, regulation of signaling, response to chemicals, regulation of cell communication, and more. The results of the KEGG analysis indicated that these targets were primarily enriched in pathways in cancer, Alzheimer’s disease, insulin resistance, prostate cancer, measles, lipid and atherosclerosis, and others (Figure 4E,F).

### 2.5. Gene Expression Omnibus (GEO) Dataset Analysis of AD-Associated MS Targets Related to Aβ and Tau Pathology

**Figure 5:**
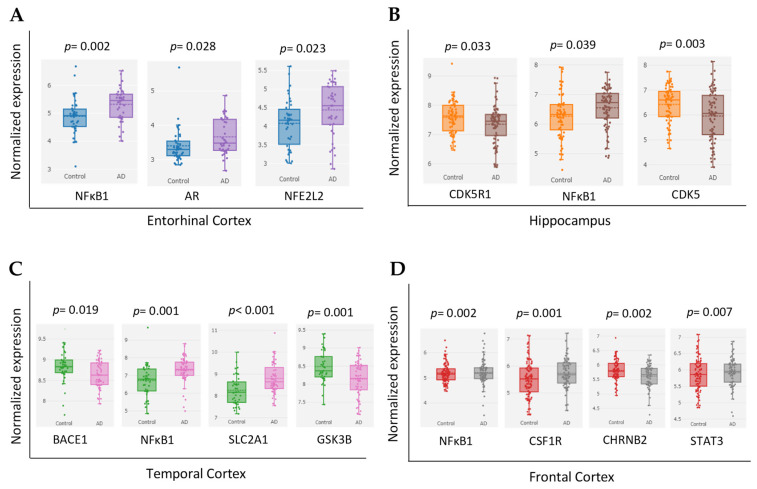
Core targets of MS in the control and AD groups of the GEO dataset. (**A**) Cross-platform normalized expression levels of NFκB1, AR, and NFE2L2 in the entorhinal cortex. (**B**) Cross-platform normalized expression levels of CDK5R1, NFκB1, and CDK5 in the hippocampus. (**C**) Cross-platform normalized expression levels of BACE1, NFκB1, SLC2A1, and GSK3B in the temporal cortex. (**D**) Cross-platform normalized expression levels of NFκB1, CSF1R, CHRNB2, and STAT3 in the frontal cortex.

According to the “Differential expression” module of the AlzData database, we analyzed the normalized expression values of MS target genes in healthy control and AD patients (Figure 5). The gene expression analysis in AD patients of the entorhinal cortex (Figure 5A), hippocampus (Figure 5B), temporal cortex (Figure 5C), and frontal cortex (Figure 5D) brain regions indicates significant dysregulation in several essential MS genes. Notably, NFKB1 is markedly upregulated in the hippocampus (FDR = 0.174) and frontal cortex (FDR = 0.008), suggesting an inflammatory response in these regions [30]. SLC2A1 exhibits substantial upregulation in the temporal cortex (log2 FC = 0.54, FDR = 0.004), potentially indicating altered glucose metabolism [31]. CSF1R, associated with microglial activation [32], shows upregulation in the frontal cortex (log2 FC = 0.28, FDR = 0.012). Furthermore, GSK3B, a critical enzyme in tau phosphorylation [33], is significantly downregulated in the temporal cortex (log2 FC = −0.35, FDR = 0.009). The results indicate that AD disrupts inflammatory, metabolic, and tau phosphorylation pathways, which play a role in the development of the disease.

### 2.6. Molecular Docking Analysis

**Figure 6:**
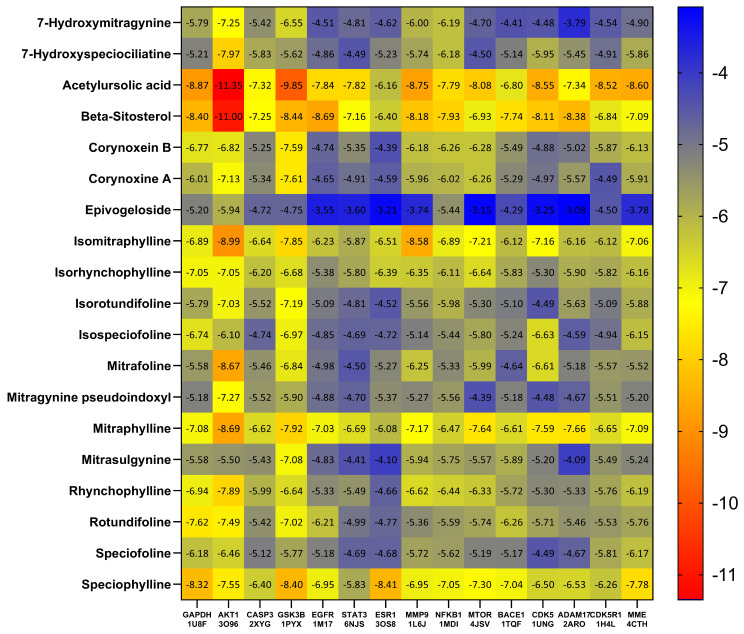
The heat map of docking score from MS and core targets (kcal/mol). Energy values were all negative, with a more negative score (red) and more binding energy.

**Figure 7:**
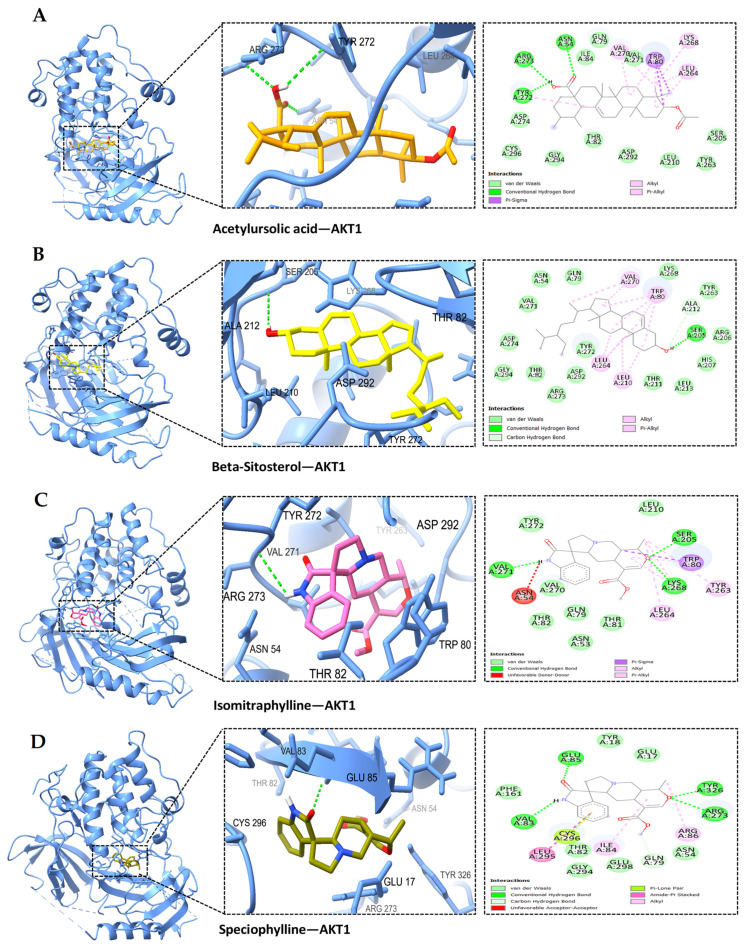
The docked complexes of AKT1 demonstrate significant binding affinities for MS compounds and hydrogen bonds: (**A**) acetylursolic acid-AKT1, (**B**) beta-sitosterol-AKT1, (**C**) isomitraphylline-AKT1, (**D**) speciophylline-AKT1. Target interaction residues are highlighted as green sticks.

**Figure 8:**
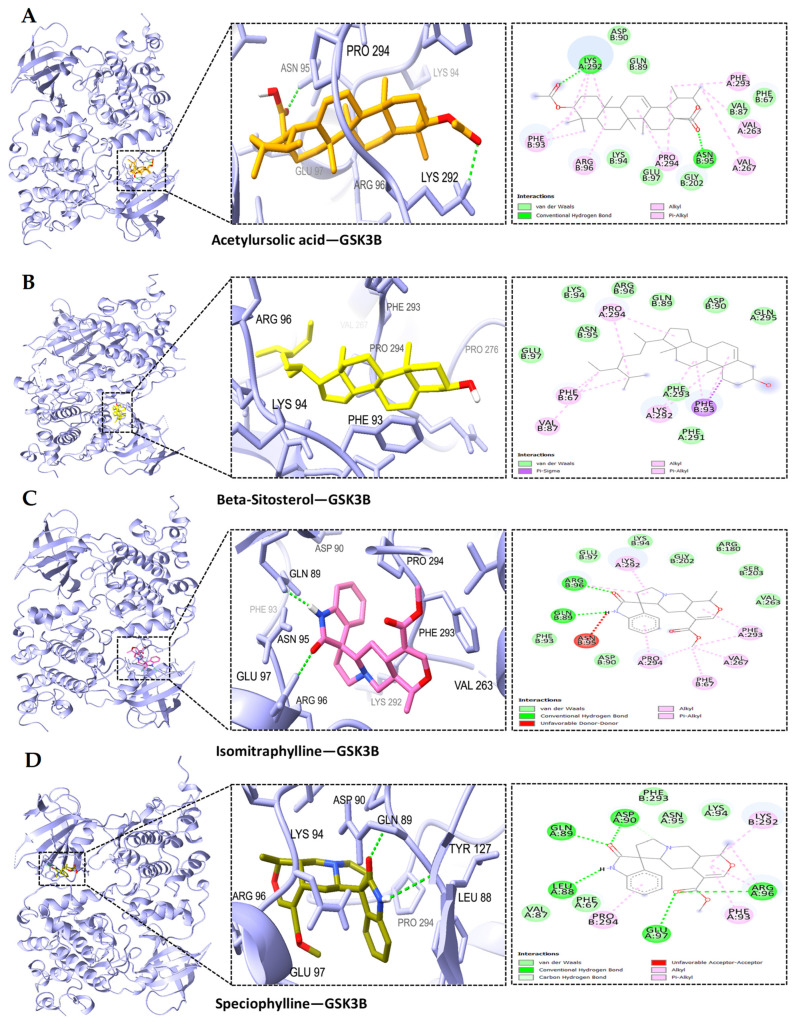
The docked complexes of GSK3B demonstrate significant binding affinities for MS compounds and hydrogen bonds: (**A**) acetylursolic acid-GSK3B, (**B**) beta-sitosterol-GSK3B, (**C**) isomitraphylline-GSK3B, (**D**) speciophylline-GSK3B. Target interaction residues are highlighted as green sticks.

**Figure 9:**
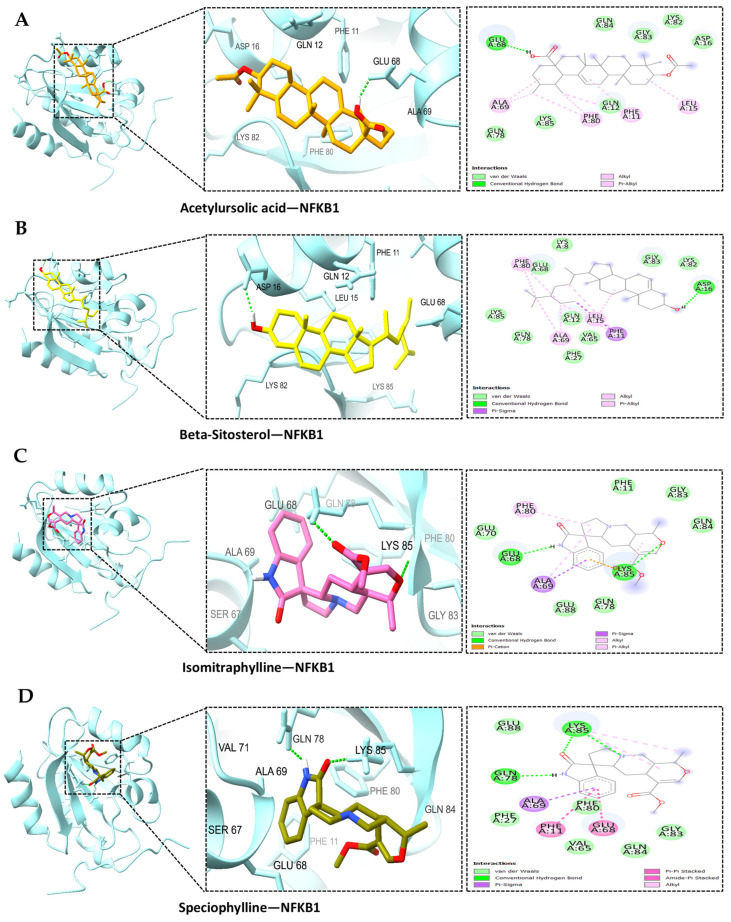
The docked complexes of NFκB1 demonstrate significant binding affinities for MS compounds and hydrogen bonds: (**A**) acetylursolic acid-NFκB1, (**B**) beta-sitosterol-NFκB1, (**C**) isomitraphylline-NFκB1, (**D**) speciophylline-NFκB1. Target interaction residues are highlighted as green sticks.

**Figure 10:**
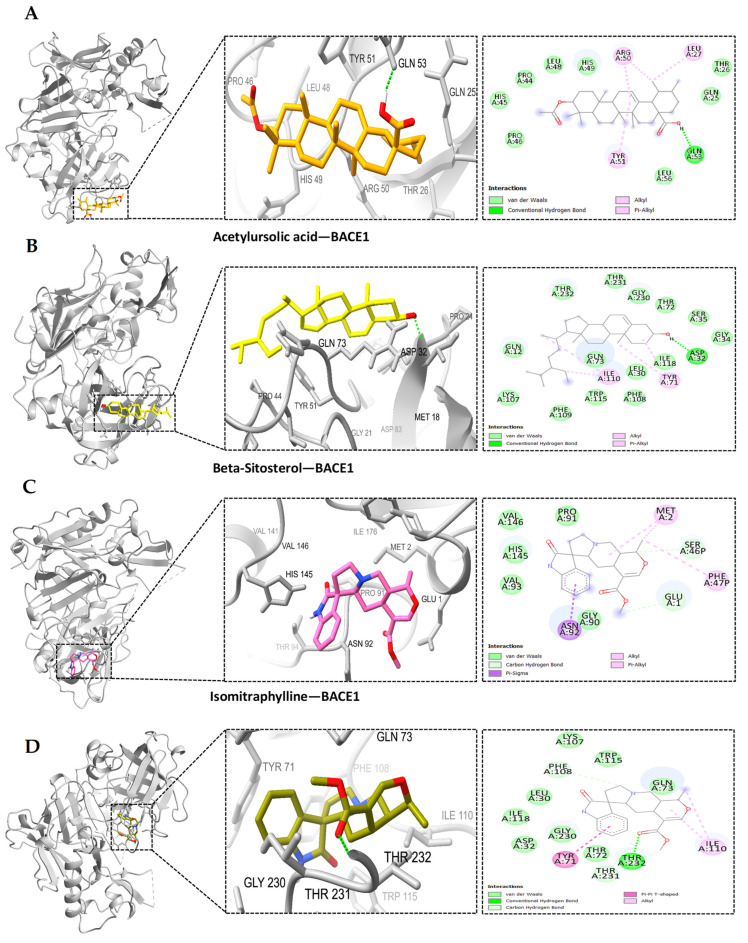
The docked complexes of BACE1 demonstrate significant binding affinities for MS compounds and hydrogen bonds: (**A**) acetylursolic Acid-BACE1, (**B**) beta-Sitosterol-BACE1, (**C**) isomitraphylline-BACE1, (**D**) speciophylline-BACE1. Target interaction residues are highlighted as green sticks.

Based on the PPI network and KEGG enrichment analysis, a set of ten hub genes (GAPDH, AKT1, CASP3, GSK3B, EGFR, STAT3, ESR1, MMP9, NFκB1, and MTOR) and five potential targets (BACE1, CDK5, ADAM17, CDK5R1, and MME) have been recognized as having involvement in the neuroprotective effects on AD-associated with MS. We conducted molecular docking simulation involving 15 genes and the MS compound based on the top-ranked poses binding energy prediction, utilizing the MOE scoring system and hydrogen bond information for the top-ranked posture. Each compound underwent docking in 10 distinct positions throughout the docking run. The docking information for the top-ranked poses (kcal/mol) is briefly presented in Figure 6. The study also found that the interactions between AD-related proteins AKT1, GSK3B, NFκB1, BACE1, and potential compounds from MS, such as acetylursolic acid, beta-sitosterol, isomitraphylline, and speciophylline, showed significant binding affinities and hydrogen bonding, as shown in Table 3, suggesting their potential as therapeutic targets. AKT1 (Figure 7A–D) strongly binds with multiple compounds, particularly acetylursolic acid, with a high docking score of −11.35 kcal/mol. Key hydrogen bonding residues for AKT1 included ASN-54, ARG-273, and TYR-272. GSK3B (Figure 8A–D) had high docking affinities with acetylursolic acid with hydrogen bonds observed at ASN-95 and LYS-292, stabilizing the interaction. NFκB1 (Figure 9A–D) demonstrated good binding with acetylursolic acid and beta-sitosterol. BACE1 (Figure 10A–D) also showed strong affinities with acetylursolic acid (−6.80 kcal/mol) and beta-sitosterol (−7.74 kcal/mol), forming hydrogen bonds with GLN-53 and ASP-32, respectively.

### 2.7. In Vitro Experimental Validation

#### 2.7.1. Total Alkaloid, Phenolic, and Flavonoid Content Assay of *M. speciosa* Extract

**Figure 11:**
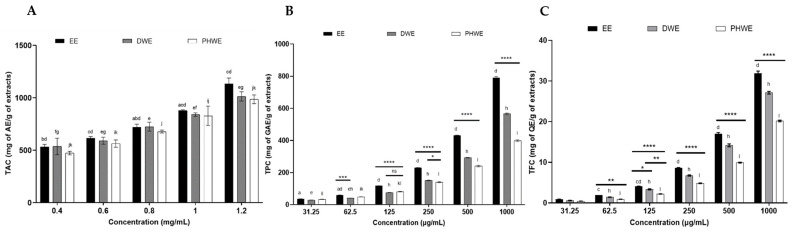
The total alkaloid, phenolic, and flavonoid contents of EE, DWE, and PHWE of *M. speciosa* leaves: (**A**) the total alkaloid content, (**B**) the total phenol content, (**C**) the total flavonoid content. The results of the experiments were expressed in mg of AE/g, GAE/g, and QE/g of the extract of the mean ± standard error of the means (SEM) (n = 3). TAC = total alkaloid content, TPC = total phenolic content, TFC = total flavonoid content, EE = ethanolic extract, DWE = distilled water extract, PHWE = pressurized hot water extract. a, b, c, d; e, f, g, h; and i, j, k, l denote comparison between the concentrations of EE, DWE, and PHWE, respectively. * denotes the comparison between EE, DWE, and PHWE. *, a, e, i, *p* < 0.05; **, b, f, j, *p* < 0.01; ***, c, g, k, *p* < 0.001; ****, d, h, l, *p* < 0.0001; ns, non-significant.

The well-known antioxidant properties of natural products, especially alkaloids and secondary metabolites like phenolics and flavonoids, play a protective role against oxidative stress and chronic degenerative diseases [34]. Alkaloids, an essential class of phytochemicals, are recognized for their neuroprotective and antioxidant properties, which may help reduce the progression of neurodegenerative diseases by protecting neurons and modulating related molecular pathways [35]. As shown in Figure 11A, among the extracts analyzed, EE had the highest total alkaloid content (TAC), measuring 1135.7 ± 53.07, followed by DWE at 1013.41 ± 43.92 and PHWE at 985.79 ± 41.56 mg AE/g at 1.2 mg/mL. These results demonstrate the superior alkaloid concentration in EE, using atropine as the standard. Phenolic compounds are recognized for their strong antioxidative solid properties, contributing directly to antioxidant activity [36]. Figure 11B shows the total phenolic content (TPC) in the three MS leaf extracts, with EE presenting the highest TPC at 789.18 ± 9.45 mg GAE/g dry weight, followed by DWE at 566.22 ± 5.48 mg GAE/g dry weight and PHWE at 399.55 ± 5.25 mg GAE/g dry weight, using gallic acid as the standard. These results correlate with previous studies that reported TPC values of 130.58 ± 0.68 mg/g for aqueous extracts and 252.92 ± 1.15 mg/g for ethanolic extracts, respectively [37]. Furthermore, additional research identified phenolic content of 88.37 ± 0.70 mg/g in alkaloid extracts and 66.00 ± 1.23 mg/g in aqueous extracts [20].

Flavonoids are vital antioxidants that neutralize ROS through scavenging actions. Combining polyphenols containing flavonoid structures with aluminum chloride produces a yellow solution that quickly turns red under alkaline conditions [36]. Figure 11C displays the total flavonoid content (TFC), indicating that EE had the highest flavonoid levels (31.87 ± 0.56 mg QE/g dry weight), while PHWE had the lowest (20.16 ± 0.19 mg QE/g dry weight), with DWE falling in between (27.13 ± 0.33 mg QE/g dry weight). The assay used quercetin as the standard, revealing that flavonoid levels were noticeably higher in organic extracts than in aqueous ones, consistent with earlier findings. Previous research noted significant TFC in ethanolic extracts (194.00 ± 5.00 QE mg/g), while other solvents such as ethyl acetate (141.50 ± 10.00 QE mg/g) and methanol (125.25 ± 1.25 QE mg/g) produced results comparable to those of aqueous extracts (115.25 ± 6.25 QE mg/g) [21].

#### 2.7.2. DPPH Scavenging Assay

**Figure 12:**
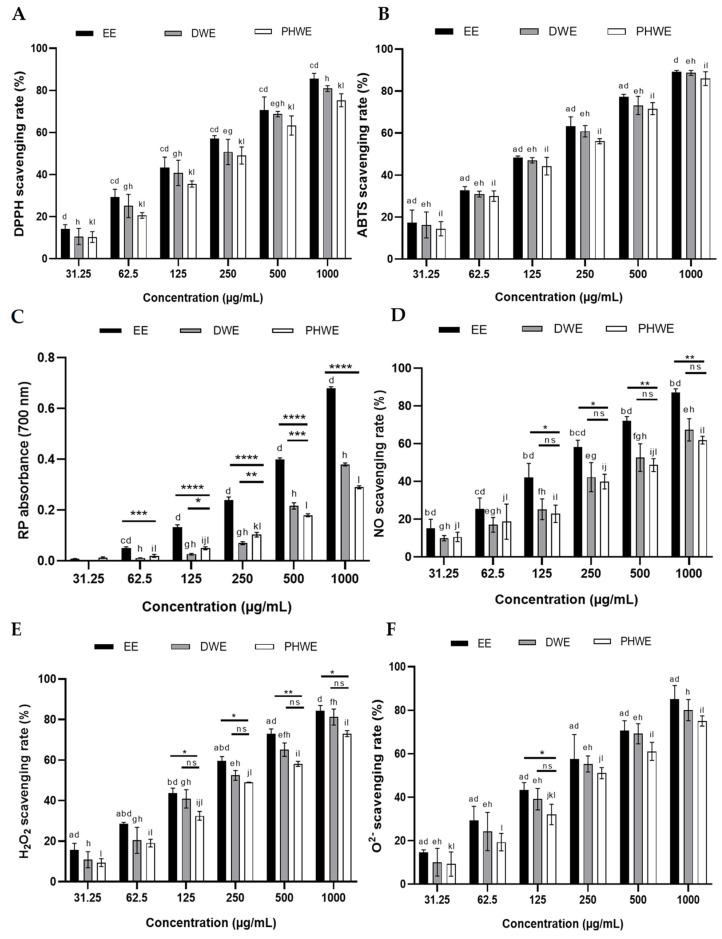
Antioxidant activities of different concentrations of EE, PHWE, and DWE (31.25–1000 μg/mL) as determined by (**A**) DPPH free radical-scavenging assay, (**B**) ABTS free radical-scavenging assay, (**C**) reducing power assay, (**D**) nitric oxide radical-scavenging assay, (**E**) hydrogen peroxide scavenging assay, and (**F**) scavenging of superoxide radical by alkaline DMSO method. The results of the experiments were expressed as the mean ± standard error of the means (SEM) (n = 3). EE = ethanolic extract, DWE = distilled water extract, PHWE = pressurized hot water extract. a, b, c, d; e, f, g, h; and i, j, k, l denote comparison between the concentrations of EE, DWE, and PHWE, respectively. * denotes the comparison between EE, DWE, and PHWE. *, a, e, i, *p* < 0.05; **, b, f, j, *p* < 0.01; ***, c, g, k, *p* < 0.001; ****, d, h, l, *p* < 0.0001; ns, non-significant.

1,1-diphenyl-2-picrylhydrazyl (DPPH) is commonly considered a model for a lipophilic radical to test the antiradical activity of antioxidants by reacting with the methanolic DPPH solution, being reduced, and transforming into a non-radical [DPPH–H] [38]. DPPH screening of plant extracts is effective for selecting antioxidants rich in radical scavengers like flavonoids [39]. Ethanol and aqueous extracts of MS showed a concentration-dependent rise in the scavenging of DPPH free radicals. EE led to higher DPPH radical scavenging activity, 85.49 ± 2.60% at 1000 µg/mL (Figure 12A), which was markedly higher than DWE and PHWE.

#### 2.7.3. ABTS Radical Scavenging Assay

In this study, the suppression of the absorbance of ABTS^+^ in a concentration-dependent manner is shown in Figure 12B. ABTS scavenging follows a similar pattern to DPPH, with EE leading in scavenging ability at around 89.18% at 1000 µg/mL. DWE and PHWE also show increased activity, though slightly less effective than EE. The scavenging of ABTS^+^ by phenolic antioxidants is observed through hydrogen atom donation, reducing the blue/green color of ABTS^+^ and evaluating scavenging activity [40]. León et al. studied the phytochemical characterization. They showed that the leaves of MS had alkaloids, flavonoid epicatechin, a saponin daucosterol, the triterpenoid saponins quinovic acid 3-O-β-D-quinovopyranoside, quinovic acid 3-O-β-D-glucopyranoside, and glycoside derivatives [41].

#### 2.7.4. Reducing Power Assay

As illustrated in Figure 12C, Fe^3+^ was transformed into Fe^2+^ in the presence of MS extract and the reference compound ascorbic acid to measure the reductive capability. RP increased significantly with concentration, particularly for EE 0.68, which showed the highest RP at 1000 µg/mL, suggesting a robust ability to donate electrons. This result indicates that maximum activity was displayed at an increased dose absorbance [42].

#### 2.7.5. Nitric Oxide Radical Scavenging Assay

The nitric oxide radical (NO•) is a short-lived, potent vasodilator that can become toxic when it reacts with oxygen or superoxide anion radicals [43]. The procedure is based on the principle that NO generated spontaneously from sodium nitroprusside in aqueous solution at physiological pH interacts with oxygen to produce nitrite ions that can be estimated using Griess reagent. EE exhibited good NO scavenging activity between 31.25 and 1000 μg/mL in a dose-dependent manner. The percentage inhibition was increased with increasing extract concentration (Figure 12D), which has been implicated in inflammation, cancer, and other pathological conditions.

#### 2.7.6. Hydrogen Peroxide Scavenging Assay

H_2_O_2_ scavenging is a vital antioxidant feature, and in the presence of electron donors, it accepts electrons and is decomposed into water [44]. MS extracts could scavenge H_2_O_2_ concentration-dependent after 10 min incubation (Figure 12E). EE demonstrates the highest H_2_O_2_ scavenging activity, reaching about 80% at the highest concentration, followed by PHWE and DWE. H_2_O_2_ is a weak oxidizing agent that inactivates a few enzymes directly, such as the glyceraldehyde-3-phosphate dehydrogenase found in the glycolytic pathway, aconitase, and a-ketoglutarate dehydrogenase found in the Krebs cycle, usually by oxidation of essential thiol (-SH) groups [45]. It can rapidly cross cell membranes, and inside, it can generate the highly reactive hydroxyl radical (HO•) by reacting with Fe^2+^ and possibly Cu^2+^ ions, which are responsible for many of its toxic effects [44].

#### 2.7.7. Scavenging of Superoxide Radical by Alkaline DMSO Method

Superoxide (O^2−^), which is less toxic, is generated in biological systems during cellular respiration [43]. It can produce incomplete metabolism oxygen damage biomolecules directly or indirectly by forming H_2_O_2_, OH•, and peroxynitrite or singlet oxygen [43,46]. Overproduction of O^2−^ radical contributes to redox imbalance and is associated with harmful physiological consequences [47]. Moderate inhibition of the O^2−^ was observed with 1000 μg/mL EE at 85.10 ± 3.59%, while DWE and PHWE showed 80.08 ± 2.82 and 75.04 ± 1.37%, at the same concentration (Figure 12F).

#### 2.7.8. SH-SY5Y Cell Viability Assay

**Figure 13:**
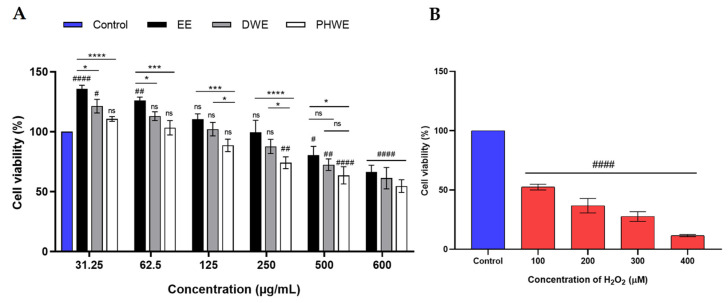
SH-SY5Y cells were treated with different concentrations of extracts and H_2_O_2_ to determine the cytotoxicity. (**A**) SH-SY5Y cells were exposed to 31.25–600 μg/mL of EE, DWE, and PHWE for 24 h, (**B**) H_2_O_2_ (100–400 μM) towards SH-SY5Y cell. The results of the experiments were expressed as the mean ± standard error of the means (SEM) (n = 3). EE = Ethanolic extract, DWE = Distilled water extract, PHWE = Pressurized hot water extract. # denotes compared to control (untreated), and * denotes the comparison between EE, DWE, and PHWE. #, *, *p* < 0.05; ##, *p* < 0.01; ***, *p* < 0.001; ####, ****, *p* < 0.0001; ns, non-significant.

The MTT assay assessed the cytotoxic impact on SH-SY5Y cells using six concentrations (31.25–600 μg/mL) of EE, DWE, and PHWE. Control cells that were not exposed to the extracts served as viability standards. The results shown in Figure 13A reveal that cell viability decreased after 24 h of treatment with the extracts as their concentrations increased. Specifically, cell viability varied from 135.68% ± 1.81% to 66.49% ± 3.24% for EE, 121.39% ± 3.29% to 61.29% ± 5.15% for DWE, and 101.67% ± 1.18% to 54.59% ± 3.05% for PHWE. Notably, the lower concentrations (31.25, 62.5, and 125 μg/mL) improved cell viability, suggesting their non-toxic properties and possible protective effects against oxidative stress. Based on these results, 31.25, 62.5, and 125 μg/mL concentrations were chosen for further investigation because they exhibited minimal cytotoxicity and promoted cell survival, indicating a protective role even at the lowest dosage that possessed the potential to alleviate oxidative stress. The neuron oxidative stress model, developed by treating SH-SY5Y cells with H_2_O_2_, has become widely utilized in studying neuronal oxidative stress [48,49]. The cells were subjected to different concentrations of H_2_O_2_ (100–400 μM) for 90 min, with viability results demonstrating a dose-dependent decline. Specifically, exposure to 100 μM H_2_O_2_ led to a 40–50% reduction in cell viability compared to the control group (Figure 13B), establishing 100 μM as the optimal concentration for forthcoming experiments.

#### 2.7.9. Neuroprotective Effects of MS Extract or Acetylursolic Acid Against H_2_O_2_-Induced SH-SY5Y Cell

**Figure 14:**
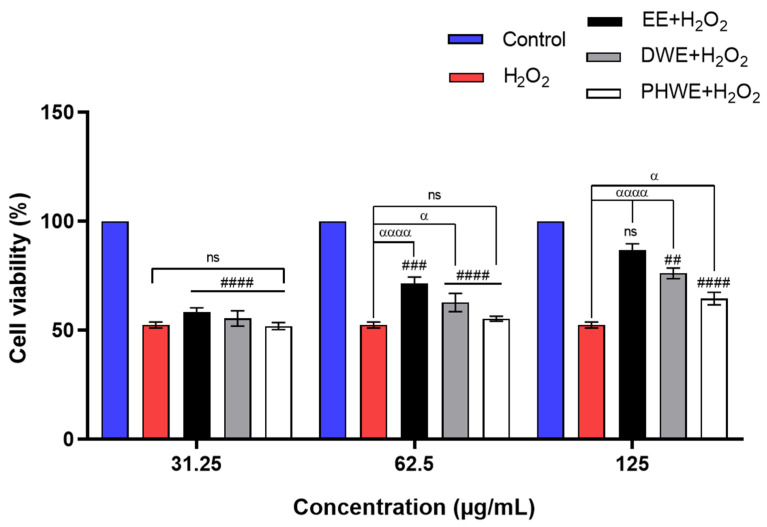
SH-SY5Y cells were treated with EE, DWE, and PHWE (31.25, 62.5, and 125 μg/mL) concentrations for 24 h after induced 100 μM H_2_O_2_. The results of the experiments were expressed as the mean ± standard error of the means (SEM) (n = 3). (α *p* < 0.05, αααα *p* < 0.0001) compared to H_2_O_2_-treated cell; (## *p* < 0.01, ### *p* < 0.001, #### *p* < 0.0001, ns: non-significant) compared to control (untreated). EE = ethanolic extract, DWE = distilled water extract, PHWE = pressurized hot water extract.

To assess the neuroprotective properties of the extracts, SH-SY5Y cells were treated with EE, DWE, and PHWE at doses of 31.25, 62.5, and 125 μg/mL for 24 h. The cells were then exposed to 100 μM H_2_O_2_ to induce oxidative stress for 2 h induction of the oxidative stress [50]. As anticipated, treatment with H_2_O_2_ led to a significant rise in reactive oxygen species ROS production compared to the control group (*p* < 0.0001). Nevertheless, post-treatment with the higher doses of EE and DWE notably reduced ROS generation, bringing levels down to 86.96 ± 2.74% and 76.14 ± 2.47%; *p* < 0.0001, respectively, compared to the H_2_O_2_-treated group. PHWE, while showing some protective effects, resulted in a less pronounced reduction in ROS levels (64.52 ± 2.88%; *p* < 0.05). These findings indicate that both EE and DWE extracts are effective neuroprotective agents capable of maintaining cellular integrity and mitigating oxidative damage caused by H_2_O_2_. In contrast, PHWE exhibited limited efficacy, underscoring the more significant bioactivity of EE and DWE (Figure 14). This study demonstrates that MS extract, mainly due to its acetylursolic acid content, holds considerable promise for therapeutic intervention in AD by targeting oxidative stress pathways and modulating critical proteins involved in neuronal survival and neuroinflammation.

## 3. Discussion

This research employs network pharmacology, molecular docking, and in vitro studies to investigate how *M. speciosa* can effectively address AD by examining its active compounds, targets, pathways, and biological mechanisms.

AD is a multifaceted brain disorder primarily characterized by the accumulation of Aβ plaques and hyperphosphorylated tau protein, resulting in neuronal harm and cognitive deterioration [51,52]. Additional factors contributing to this condition include neuroinflammation and oxidative stress [53,54]. As the most common type of dementia, AD represents a significant global health concern, especially in light of increasing healthcare expenses and an aging demographic [54]. Current therapies may relieve symptoms but fail to halt neurodegeneration, highlighting the necessity for innovative treatments [15].

*M. speciosa* contains 79 secondary metabolites, which include numerous alkaloids, flavonoids, and polyphenols, showing a dual profile characterized by potential abuse and toxicity alongside notable therapeutic properties [18,47]. It is particularly abundant in polyphenolic compounds recognized for their antioxidant properties, essential in mitigating oxidative stress, a key element in AD [55,56]. Our hypothesis suggested that MS could safeguard SH-SY5Y cells against oxidative harm induced by H_2_O_2_ at lower concentrations. We observed that treatments with extracts like EE, DWE, and PHWE reduced cell death dose-dependently. EE demonstrates significant antioxidant activity due to its rich content of polyphenols, alkaloids, and flavonoids, which help reduce oxidative stress and inflammation by modulating Nrf2 signaling and scavenging free radicals [57]. However, higher concentrations can be cytotoxic, emphasizing the need for caution.

Acetylursolic acid, a derivative of ursolic acid, is gaining attention for its cognitive advantages, especially in memory enhancement and neuroprotection [58]. It has been shown to improve learning and memory deficits caused by neurotoxins such as Aβ, which are associated with AD [59]. Its impacts include increasing neuronal density by upregulating growth-associated protein GAP43, boosting the ubiquitin–proteasome system (UPS) to eliminate damaged proteins, and reducing neuroinflammation by decreasing pro-inflammatory cytokines like IL-1β, IL-6, and TNF-α [58,60]. By inhibiting pathways such as NF-κB signaling, acetylursolic acid alleviates neuroinflammation, fostering a neural environment that supports cognitive function [26]. Ursolic acid also provides neuroprotective effects through its antioxidant capabilities, lowering oxidative stress markers such as malondialdehyde (MDA) while raising glutathione (GSH) levels [61]. It lessens Aβ-induced neurotoxicity and activates the Nrf2 pathway for antioxidant protection, promoting proteins like heme oxygenase-1 (HO-1) and superoxide dismutase (SOD) to combat oxidative damage [62,63].

Beta-sitosterol, a widely occurring phytosterol resembling cholesterol in structure, is promising as a potential treatment for AD due to its neuroprotective properties [64]. Research in APP/PS1 transgenic mice indicates it can enhance cognitive performance by improving spatial learning, decreasing Aβ accumulation, and restoring dendritic spine density [65]. One significant mechanism involves its ability to inhibit cholinesterase, which raises acetylcholine levels by blocking acetylcholinesterase (AChE) and butyrylcholinesterase (BChE), thereby improving learning and memory [66]. Additionally, beta-sitosterol promotes the expression of nerve growth factor (NGF), which aids neuronal survival and combats neurodegenerative changes associated with impaired NGF signaling in AD [67]. Its benefits include stimulating NGF activity, inhibiting β- and γ-secretase activity connected to elevated cholesterol, and lowering oxidative stress [68]. By stabilizing cell membranes, it enhances receptor signaling and cellular communication, and it can cross the blood–brain barrier (BBB) to offer direct neuroprotective effects [69].

Isomitraphylline, an oxindole alkaloid derived from the Mitragyna plant, shows potential in AD research due to its neuroprotective, anti-inflammatory, and antioxidant effects [18]. It inhibits Aβ aggregation by influencing BACE1 activity and promotes autophagy for clearing harmful proteins, significantly decreasing cytotoxicity in neuroblastoma cells and lowering oxidative stress levels [70,71]. In vitro studies revealed that at a concentration of 50 μM, isomitraphylline inhibited Aβ aggregation by roughly 60.32% ± 2.61% [70]. In neuroblastoma SH-SY5Y cells exposed to Aβ or H_2_O_2_, isomitraphylline displayed protective properties, particularly at concentrations of 10 μM and 20 μM, reducing cytotoxicity caused by these agents [70,71]. The compound also helped preserve mitochondrial membrane potential, suggesting a safeguarding effect on mitochondrial function [72]. Isomitraphylline activates the Nrf2 pathway, boosting antioxidant enzymes and diminishing oxidative damage [73]. The GSK3 family includes GSK3α and GSK3β, with GSK3β predominantly found in the brain, while GSK3α is notably present in regions like the hippocampus, cerebral cortex, striatum, and cerebellum; GSK3β is found throughout nearly all brain areas [74]. Furthermore, isomitraphylline inhibits the NF-κB signaling pathway to reduce neuroinflammation and supports neuronal wellbeing by blocking GSK-3β [71].

Speciophylline decreases ROS in neuroblastoma cells subjected to Aβ, safeguarding against oxidative stress by inhibiting the NF-κB pathway [75]. Its anti-inflammatory characteristics help alleviate microglial activation and cytokine production, lessening the impact of chronic inflammation on neurons [18]. Speciophylline promotes cell survival by engaging the PI3K/Akt pathway, inhibiting GSK-3β, and reducing tau hyperphosphorylation [71]. Additionally, it inhibits BACE1 activity to lower Aβ production and sustain synaptic functions essential for memory [76]. By downregulating mTOR, it promotes autophagy and increases BDNF expression, which is vital for neurogenesis and synaptic plasticity necessary for cognitive function in AD [77].

The resulting network showcased the capacity of bioactive compounds to influence AD through complex interactions involving 60 proteins across various pathways. Through PPI analysis, we pinpointed 60 nodes and 470 edges. The enzyme-modifying proteins include GSK3B, TBK1, CASP7, PTK2B, F2, APH1B, PSEN1, AKT1, ADAM17, MTOR, BACE1, PSEN2, CDK5, FYN, ADAM10, CTSD, CASP3, and the metalloproteinases MME, ECE1, and MMP9. Core targets identified include GAPDH, AKT1, CASP3, GSK3B, EGFR, STAT3, ESR1, MMP9, NFκB1, and MTOR, which are significant in AD progression. Further analysis through GO and KEGG highlighted numerous pathways, emphasizing connections with other diseases relating to the genes, as mentioned earlier. The identified GO molecular functions included catalytic activity, enzyme binding, aspartic-type endopeptidase activity, protein binding, identical protein binding, endopeptidase activity, and protein kinase activity. Moreover, the KEGG pathway analysis emphasizes the crucial role of AD pathways within the specified network. This finding supports the idea that MS could be a promising candidate for AD treatment. Beyond the established AD pathways, our research also indicated involvement in cancer pathways. Collectively, these results suggest a hopeful, multifaceted strategy for MS as a potential multitarget therapeutic agent. Additionally, we found 20 targets associated with Aβ and tau pathology when examining gene expression in the entorhinal cortex, hippocampus, temporal cortex, and frontal cortex of AD patients. The results from the molecular docking analysis indicated a range of binding free energies for the docked ligands, with values from −5.50 to −11.35 kcal/mol for RAC-alpha serine/threonine-protein kinase (AKT1), −4.75 to −9.85 kcal/mol for Glycogen synthase kinase-3 beta (GSK3B), −5.33 to −7.93 kcal/mol for the Nuclear factor NF-kappa-B p105 subunit (NFκB1), and −4.29 to −7.74 kcal/mol for beta-secretase 1 (BACE1). The observed binding interactions between the bioactive compounds and target proteins resulted from a complex interplay of hydrophobic interactions, the formation of hydrogen bonds, and other essential interactions such as π-stacking and salt bridge formation. Our data indicated that acetylursolic acid, beta-sitosterol, isomitraphylline, and speciophylline exhibited the highest affinity for all the receptors above in the experimental series. The superior binding strength of acetylursolic acid can be attributed to its extensive interactions with a larger number of essential amino acid residues. Overall, comprehension and optimization of these interactions are critical for crafting potent and selective drug candidates in molecular docking studies. A limitation of the research is that the predicted target information for bioactive ingredients and diseases was sourced from databases, necessitating regular updates to maintain data accuracy. Nonetheless, employing a data mining strategy, our study offers insights into potential molecular mechanisms for the effects of MS on neuroprotection that may aid in preventing AD.

**Figure 15:**
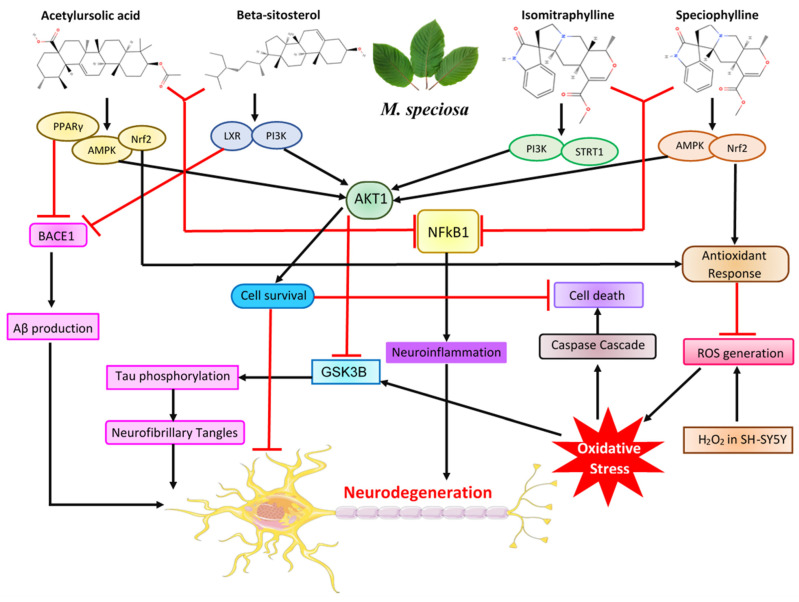
The constituents of *M. speciosa* interact with multiple targets and pathways to inhibit neurodegeneration.

In conclusion, this research used network pharmacology, molecular docking, and in vitro SH-SY5Y cell experiments to study how *M. speciosa* can prevent and treat AD. It found that multiple components of *M. speciosa* work through various targets and pathways (Figure 15), providing a foundation for further exploration of its mechanisms and clinical applications in AD prevention and treatment.

## 4. Materials and Methods

### 4.1. Chemicals and Reagents

The current study employed chemicals and reagents of analytical grade, including bromocresol green (BCG), chloroform (CHCl_3_), Folin–Ciocalteu reagent, sodium carbonate (Na_2_CO_3_), gallic acid, ethanol, quercetin, aluminum chloride (AlCl_3_), ascorbic acid, methanol, 2,2-Diphenyl-1-picrylhydrazyl (DPPH), 2,2′-azino-bis(3-ethylbenzothiazoline-6-sulfonic acid) (ABTS), potassium persulfate (K_2_S_2_O_8_), dimethyl sulfoxide (DMSO), hydrogen peroxide (H_2_O_2_), nitro blue tetrazolium (NBT), potassium ferricyanide (K_3_[Fe(CN)_6_]), trichloroacetic acid, ferric chloride (FeC1_3_), sodium nitroprusside (SNP), and Griess reagent was procured from Sigma-Aldrich, St. Louis, MO, USA. For cell assessment, Dulbecco’s modified Eagle’s medium (DMEM)/Ham’s F-12, fetal bovine serum (FBS), penicillin or streptomycin, phosphate-buffer saline (PBS) (pH 7.4), 3-(4,5-dimethylthiazol-2-yl)-2,5-diphenyltetrazolium bromide (MTT) powder, trypsin-EDTA (0.25% trypsin, 1 mM EDTA), and trypan blue stain (0.4%) were utilized from Life Technologies Corporation, Gibco, Grand Island, NY, USA.

### 4.2. Plant Materials Collection

Fresh leaves of MS were collected from an agroforestry farm in Huasai, Nakhon Si Thammarat Province, Thailand, and the plant specimen was identified at the Faculty of Traditional Thai Medicine, Walailak University. The leaves were carefully washed with tap water and sliced into smaller cuts and the fresh weight was recorded. The fresh leaves were then ground (1.0 mm) into a fine powder with a laboratory grinder (WF-20B, 220–240 V, 3000 W, 50–60 Hz, n25,000 r/min, thaigrinder, Thailand). The powder was weighed as a wet weight before being transferred into a sealed jar ready to be used for extract preparation. The yield extraction of each extract was calculated by using the equation,

(1) $$Percentage\;yield\;\left(\%\right)=\frac{weight\;of\;crude\;extract}{weight\;of\;dry\;sample}\times 100$$

### 4.3. Extract Preparation

The preparation of the DWE, PHWE, and EE of MS leaves was carried out by Parthasarathy et al. [20] with slight modifications. For the preparation of DWE, MS leaves powder (2000 g) (1:10) was macerated with distilled water (20 L) for 72 h at room temperature. The mixture was aliquoted into Duran bottles (1 L/bottle) with occasional stirring. For PHWE, leaves powder was mixed with distilled water for a total of (2800 g in 28 L) in Duran bottles and placed in an autoclave at 12 °C, 15 psi for 1 h, and the method for EE, 2700 g of leaves powder soaked in 2.5 L 99.9% ethanol in Duran bottles for 72 h at room temperature (26 to 28 °C) while stirring occasionally. Then, boiled mixture was left to cool, and all the mixture was filtered with a stainless-steel wire maze filter 45 μm (325 No.) (Anping Yuansheng Mesh Co., Ltd. Hengshui, China) and then centrifuged (Hettich, Rotina 420 R, Germany) at 4000 rpm for 10 min, again filtered the supernatant with Whatman filter paper No. 1 (Sigma-Aldrich, 125 mm diameter) individually. The crude water extract of DWE (100 g) and PHWE (150 g) was pre-frozen (Eyela PFR-1000, Japan) at −40 °C for 15 min and lyophilized (Eyela FDU-2100, Japan) at −80 °C for 24 h. The lyophilized powder was collected and stored at −20 °C until analysis and coded as ‘DWE’ and ‘PHWE’. The extracted solution of EE was evaporated using a rotatory evaporator (Eyela CA-1112CE, N-1200B, A-10005, OSB-2100) system at 15 °C. Throughout the extraction process, the weight of the fresh and dry herbs and the dry extracts (88.07 g) was recorded for ‘EE.’ The yield of the extract was found to be 3.26% *w*/*w* (EE), 5.00% *w*/*w* (DWE), and 5.35% *w*/*w* (PHWE).

### 4.4. Computation Analysis

#### 4.4.1. Acquisition of Target Information and Screening of Key Targets of *M. speciosa*

A comprehensive literature review was performed using databases like PubMed and Google Scholar to discover the main phytochemicals present in MS, focusing on the keywords “*Mitragyna speciosa*” and “Kratom” [37,78,79,80,81]. Additional data were sourced from Dr. Duke’s Phytochemical and Ethnobotanical Databases [82] (https://phytochem.nal.usda.gov/), accessed on 5 September 2024 and KNApSAcK: A Comprehensive Species-Metabolite Relationship Database [83] (http://www.knapsackfamily.com/knapsack_core/top.php), accessed on 5 September 2024. For the analysis of absorption, distribution, metabolism, and excretion (ADME), Lipinski’s Rule of Five (RO5) was utilized via the SwissADME online server [84] (http://www.swissadme.ch/index.php), accessed on 5 September 2024. Compounds that violated RO5 three or more times were excluded. Target genes were identified by entering the selected compounds into the Swiss Target Prediction tool [85] (http://swisstargetprediction.ch/), accessed on 6 September 2024, with *Homo sapiens* specified as the chosen species. Additionally, compound toxicity was forecasted using the Protox platform [86] (https://tox.charite.de/protox3/), accessed on 6 September 2024, ruling out compounds with three or more violations regarding toxicity endpoints (hepatotoxicity, carcinogenicity, immunotoxicity, cytotoxicity, and mutagenicity). The target genes obtained from various sources were compiled, and duplicates were eliminated.

#### 4.4.2. Identification of Potential Targets for AD

The GeneCards [87] (https://www.genecards.org/), accessed on 15 September 2024, Online Mendelian Inheritance in Man (OMIM) [88] (https://omim.org/) accessed on 15 September 2024, and DisGeNET [89] (https://www.disgenet.org/), accessed on 15 September 2024, databases were utilized to find potential targets for AD with the keyword “Alzheimer’s disease”.

#### 4.4.3. Construction and Analysis of the PPI Network

A Venn diagram tool [90] (http://bioinformatics.psb.ugent.be/webtools/Venn/), accessed on 15 September 2024, was used to identify overlapping target genes. Common targets were then submitted to the STRING 11.0 database [91] (https://string-db.org/), accessed on 15 September 2024, for the construction of a PPI network for *Homo sapiens*. The PPI network data were imported into Cytoscape 3.4.0 [92] (http://chianti.ucsd.edu/cytoscape-3.4.0/), accessed on 15 September 2024, for visualization and analysis. Key targets were identified using the CentiScaPe 2.2 plugin in Cytoscape, which selected targets with the above-median values for degree centrality, closeness centrality, and betweenness centrality. The PPI network was saved in the TSV format and re-imported into Cytoscape 3.8.0 for additional analysis. Target proteins were classified with the Panther Classification System [93] (https://pantherdb.org/), accessed on 15 September 2024.

#### 4.4.4. GO and KEGG Pathway Annotation

GO and KEGG pathway enrichment analyses were performed using ShinyGO 0.80 [94] (http://bioinformatics.sdstate.edu/), accessed on 15 September 2024. The analysis was conducted with the species “*Homo sapiens*,”. The top 20 significant pathways were displayed in a lollipop chart and filtered for a false discovery rate (FDR) of <0.05.

#### 4.4.5. Validation of Target Changes in AD Signaling Pathway Using the AlzData Database

The AlzData database [29] (http://www.alzdata.org/), accessed on 15 September 2024, provided gene expression profiles from the brain tissues of 684 AD patients and 562 controls. Gene symbols for AD target proteins were entered into AlzData to analyze their correlation with AD pathology markers, such as Aβ and tau, to assess any target changes within critical signaling pathways.

#### 4.4.6. Prediction of Binding Affinity Between Active Components and Potential Targets of *M. speciosa* Through Molecular Docking

The 3D structures of active compounds (in SDF format) were retrieved from the PubChem database [95] (https://pubchem.ncbi.nlm.nih.gov/), accessd on 15 September 2024, while the 3D structures of target proteins (in PDB format) were sourced from the Protein Data Bank (PDB) [96] (http://www.rcsb.org), accessd on 15 September 2024. Refinements were carried out using ChimeraX software [97] (https://www.cgl.ucsf.edu/chimera/), accessd on 15 September 2024. Target structures underwent optimization and preparation for docking studies with Discovery Studio Visualizer 2020. Molecular docking simulations were conducted using AutoDock Vina 1.2.0 [98] (https://autodock.scripps.edu/), accessd on 15 September 2024, establishing grid boxes for each target protein to include all active binding sites and essential residues. The grid center coordinates and dimensions for each protein were specified according to their specific requirements, e.g., (size_x = 6.29 Å, size_y = −10.759 Å, and size_z = 19.651 Å) for (PDB: 3O96), (size_x = 22.371 Å, size_y = −0.299 Å, and size_z = 21.427 Å) for (PDB: 1PYX), (size_x = 2.064 Å, size_y = 0.687 Å, and size_z = 0.086 Å) for (PDB: 1MDI), and (size_x = 30.127 Å, size_y = 32.192 Å, and size_z = 10.381 Å) for (PDB: 1TQF) (e.g., PDB: 3O96, 1PYX, 1MDI, 1TQF). Ligands and targets were docked to assess binding affinity, shedding light on potential interactions between the compounds of MS and AD-related proteins.

### 4.5. Phytochemical and Antioxidant Analysis of M. speciosa Extracts

#### 4.5.1. Qualitative Alkaloid Analysis

Alkaloid presence in MS extracts (EE, DWE, HPWE) was confirmed using Mayer’s reagent. A white creamy precipitate indicated alkaloid presence due to the interaction with potassium mercuric iodide in Mayer’s reagent.

#### 4.5.2. Total Alkaloid Content

The TAC of MS extracts was measured using the method described by Shamsa et al. [99]. A bromocresol green (BCG) solution was created by dissolving 0.15 g of BCG in 150 mL of distilled water. Solutions of standard atropine, EE, DWE, and HPWE (0.4, 0.6, 0.8, 1, and 1.2 mL) were mixed with 5 mL of phosphate buffer (pH 4.7) and 5 mL of BCG, then shaken with 1–4 mL of chloroform. The chloroform layer containing the alkaloid-BCG complex was extracted and diluted to 10 mL, and the absorbance was measured at 470 nm using a UV-visible spectrometer (Multiskan Sky High, Thermoscientific, Singapore). TAC was calculated as atropine equivalents (AE) in mg/g of dry extract using the following equation:

(2) $$Total\;alkaoid\;content\;(TAC)=AE\times \frac{V}{m}$$

where AE is the Atropine Equivalent (mg/mL) or concentration of atropine established from the calibration curve, V is the volume of extract (mL), and m is the weight of extracts (g).

#### 4.5.3. Total Phenolic Content Assay

TPC was evaluated using a modified Folin–Ciocalteu method [100]. Extracts were diluted to a concentration of 1 mg/mL, and 20 µL from each was combined with 100 µL of 7% Folin–Ciocalteu reagent and 80 µL of 10% Na_2_CO_3_. Following a 30 min incubation at 45 °C, absorbance readings were taken at 765 nm. Gallic acid served as a standard (31.25–1000 µg/mL), and TPC was determined as gallic acid equivalents (GAE) in mg/g of dry extract using the following equation:

(3) $$Total\;phenolic\;content\;(TPC)=GAE\times \frac{V}{m}$$

where GAE is the Gallic Acid Equivalence (mg/mL) or concentration of gallic acid established from the calibration curve, V is the volume of extract (mL), and m is the weight of extracts (g).

#### 4.5.4. Total Flavonoid Content Assay

TFC was determined using a colorimetric method with aluminum chloride [101] colorimetric assay by Wairata et al. A mixture of 100 µL of extract (1 mg/mL) and 100 µL of 2% AlCl_3_ solution was allowed to incubate at room temperature for 30 min. The absorbance was recorded at 415 nm, with quercetin serving as the standard. TFC was expressed as quercetin equivalents (QE) in mg/g of dry extract, calculated by the following equation:

(4) $$Total\;flavonoid\;content\;(TFC)=QE\times \frac{V}{m}$$

where QE is the Quercetin Equivalence (mg/mL) or concentration of quercetin solution established from the calibration curve, V is the volume of extract (mL), and m is the weight of the extract (g).

#### 4.5.5. DPPH Scavenging Assay

The DPPH radical scavenging activity was assessed following the method outlined by Bahadori et al. [102]. Various extract concentrations (31.25 to 1000 µg/mL) were examined using a 0.1 mM DPPH solution. Absorbance readings were taken at 517 nm after a 30 min incubation period. IC_50_ values were derived from linear regression analysis of the plotted data. The scavenging activity was calculated using the following equation:

(5) $$Scavenging\;activity\;(\%)=\frac{\left(Ao-Ae\right)}{Ao}\times 100$$

where Ao = absorbance of the blank solution and Ae = absorbance of the test (extract) solution or the standard (ascorbic acid).

#### 4.5.6. ABTS Radical Scavenging Assay

ABTS activity was assessed using the method described by Re et al. [103]. After the ABTS radical solution was prepared, 20 µL samples of ascorbic acid were combined with 180 µL ABTS solution. The mixture was measured at 734 nm after 45 min in the dark at room temperature. The percentage of ABTS inhibition was determined according to Equation (5).

#### 4.5.7. Reducing Power Assay

The reducing power of the extracts was evaluated using the method described by Bouabid et al. [104]. The extracts were mixed with phosphate buffer and K_3_[Fe(CN)_6_], then incubated at 50 °C for 30 min. Following centrifugation, the supernatant was combined with FeCl_3_ and measured at a wavelength of 700 nm. A higher absorbance reflected a more potent antioxidant activity. All tests were performed three times.

#### 4.5.8. Nitric Oxide Radical Scavenging Assay

NO is generated from aqueous SNP solution at physiological pH (7.2) by interacting with oxygen to produce stable products nitrate and nitrite ions under aerobic conditions, which the Griess Illosvoy reaction may quantify [105]. NO scavenging assay was measured using the method described by Shukla, Mehta et al. [38]. The reaction mixture contained 1 mL SNP (10 mM *w*/*v*) in phosphate buffer (pH 7.4) was added to 0.5 mL of extract (31.25–1000 μg/mL) and incubated at room temperature for 4 h. After the incubation period, 0.25 mL of Griess reagent was added. The absorbance of the pink chromophore formed during the diazotization of nitrite with the sulphanilamide and its subsequent coupling with NED was read at 546 nm. Ascorbic acid was used as standard, and ethanol was used as negative control. The percentage of nitric oxide-scavenging activity was calculated by Equation (5).

#### 4.5.9. Hydrogen Peroxide Scavenging Assay

The ability of the extract to scavenge hydrogen peroxide assay was determined according to the method of Ruch et al. [106] with minor changes. Briefly, a solution of H_2_O_2_ (40 mM) was prepared in phosphate buffer (50 mM, pH 7.4). Various concentrations of sample extracts were mixed (1:1 *v/v*) with 0.6 mL H_2_O_2_ and incubated for 10 min at room temperature. After that, absorbance was measured at 230 nm.

#### 4.5.10. Scavenging of Superoxide Radical by Alkaline DMSO Method

Superoxide scavenger inhibits the formation of a red dye formazan. The ability of the extracts to scavenge superoxide radicals by alkaline DMSO was followed by Ganapaty et al. [107]. To the reaction mixture containing 30 μL of NBT (1 mg/mL solution in DMSO) and 40 μL of the various extract concentrations, 130 μL of alkaline DMSO was added. After that, the absorbance was measured at 560 nm against the control mixture of 20 μL of distilled water with 130 μL of an alkaline DMSO solution.

### 4.6. Cell Culture

Human neuroblastoma SH-SY5Y (ATCC HTB-11) cells were purchased from Biomedia, Thailand Co., Ltd. Cells were grown in DMEM supplemented with 10% (*v/v*) FBS and 1% penicillin/ streptomycin at 37 °C under 5% CO_2_ in air (The Thermo Scientific™ Heracell™ VIOS 160i, Langenselbold, Hesse, Germany) and 95% humidity. The medium was changed every three days for continued propagation of the cells. The cells were grown until reaching approximately 80–90% confluence and then split or plated. For cell passage, the cell culture medium was removed, and the cells were washed with prewarmed PBS 1×, 2 mL of 0.25% trypsin-EDTA were added, and the cells were kept in the incubator for approximately 5 min at 5% CO_2_, 37 °C. After that, 10 mL of DMEM containing 10% FBS was added, and the cells were centrifuged (Hettich, Rotina 420 R, Kirchlengern, Germany) at 2500 rpm for 5 min. The supernatant was then removed, and the cells were resuspended in a cell culture medium and split into 96-multiwell plates at 10,000 cells/well.

#### Cell Viability Assay

The cell viability of SH-SY5Y cells was determined by the conventional colorimetric MTT reduction assay based on converting MTT to purple formazan crystals by mitochondrial dehydrogenase [108]. Initially, cells were treated with increasing concentrations of EE, DWE, and PHWE (31.25–600 μM) along with H_2_O_2_ (100, 200, 300, 400 μM) to optimize the LD_50_ value. Before treatment with EE, PHWE, DWE, and H_2_O_2,_ the cells were incubated for 4 h at 37 °C with a mixture of 2% FBS and MTT (1:1) at a 0.5 mg/mL concentration. Then, 80 μL medium solution was removed, and the cells were suspended in 20 μL of DMSO. The absorbance of formazan reduction was recorded at 570 nm to evaluate the effects of EE, DWE, and PHWE on H_2_O_2_-treated SH-SY5Y cells after 2 h, with the extracts administered to the cells for 24 h.

### 4.7. Statistical Analysis

The data were conveyed as the average ± standard error mean (SEM) based on three independent experiments. Data were first analyzed using GraphPad Prism 9.3.1 (GraphPad Software, Inc., San Diego, CA, USA) for statistical assessments. Group comparisons were conducted using one-way (ANOVA) with the Bonferroni posthoc test, and significance was determined at a *p*-value < 0.05. The comparative analysis of phytochemicals among the groups was conducted utilizing a two-way ANOVA method.

## 5. Conclusions

In this research, we adopted a thorough strategy that combined network pharmacology with extensive database exploration to pinpoint essential target proteins linked to MS that could enhance AD treatment. Our results emphasized that MS mainly involves catalytic function, enzyme interaction, and AD-related pathways, affecting vital proteins such as AKT1, GSK3B, NFκB1, and BACE1, which play significant roles in AD therapy. In vitro studies revealed that the ethanolic extract of MS exhibits superior antioxidant capabilities compared to other water-based extracts, significantly lowering ROS levels to 86.96 ± 2.74% and 76.14 ± 2.47%; *p* < 0.0001 for higher doses of EE and DWE, respectively, in comparison to the H_2_O_2_-treated group. Although PHWE displayed some protective qualities, its ROS reduction was less significant (64.52 ± 2.88%; *p* < 0.05). These findings indicate that EE and DWE are effective neuroprotective agents that can alleviate oxidative damage and maintain cellular integrity. Overall, the results imply their potential as innovative pharmacological approaches for AD, necessitating further investigation to develop improved treatment strategies.

## 6. Future Perspective

The research explored the neuroprotective benefits of *M. speciosa*, emphasizing the need for in vitro studies and advancements in network pharmacology and molecular docking to understand its effects on AD. Future research will use transgenic animal models, like 5xFAD mice [109], for behavioral assessments (e.g., Morris water maze, novel object recognition) and biochemical analyses (e.g., Aβ-plaque quantification) to gauge therapeutic effectiveness [110]. Clinical trials are essential for evaluating safety and efficacy in humans.

## Figures and Tables

**Table 1:** The compounds present in MS with the drug-likeness and toxicity predicted through SwissADME and Protox II.

Chem CID	Compound Name	MF	MW (g/mol)	Drug-Likeness					Toxicity
				HBA	HBD	LogP	BBB	BA	EP (Class)
44301524	7-Hydroxymitragynine	C_23_H_30_N_2_O_5_	414.49	7	1	2.27	No	0.55	C (IV)
275301973	7-Hydroxyspeciociliatine	C_23_H_30_N_2_O_5_	414.49	7	1	2.27	No	0.55	C (IV)
6475119	Acetylursolic acid	C_32_H_50_O_4_	498.74	4	1	7.92	No	0.85	I (V)
222284	Beta-Sitosterol	C_29_H_50_O	414.71	1	1	8.45	No	0.55	I (IV)
10475115	Corynoxine A	C_22_H_28_N_2_O_4_	384.47	5	1	2.31	Yes	0.55	C (IV)
10091424	Corynoxein B	C_22_H_28_N_2_O_4_	384.47	5	1	2.31	Yes	0.55	C (IV)
14192590	Epivogeloside	C_17_H_24_O_10_	388.37	10	4	−0.91	No	0.56	I (IV)
11726520	Isomitraphylline	C_21_H_24_N_2_O_4_	368.43	5	1	1.62	No	0.55	C (IV)
3037048	Isorhynchophylline	C_22_H_28_N_2_O_4_	384.47	5	1	2.31	Yes	0.55	C (IV)
102232176	Isorotundifoline	C_22_H_28_N_2_O_5_	400.47	6	2	1.95	No	0.55	(IV)
101289836	Isospeciofoline	C_22_H_28_N_2_O_5_	400.47	6	2	1.95	No	0.55	(IV)
5379743	Mitrafoline	C_22_H_28_N_2_O_5_	400.47	6	2	1.95	No	0.55	(IV)
44301701	Mitragynine pseudoindoxyl	C_23_H_30_N_2_O_5_	414.49	6	1	3	No	0.55	C (IV)
94160	Mitraphylline	C_21_H_24_N_2_O_4_	368.43	5	1	1.62	No	0.55	C (IV)
275540045	Mitrasulgynine	C_23_H_35_N_2_O_5_S^+^	451.6	5	1	3	No	0.55	C (IV)
5281408	Rhynchophylline	C_22_H_28_N_2_O_4_	384.47	5	1	2.31	Yes	0.55	C (IV)
5321000	Rotundifoline	C_22_H_28_N_2_O_5_	400.47	6	2	1.95	No	0.55	(IV)
5379742	Speciofoline	C_22_H_28_N_2_O_5_	400.47	6	2	1.95	No	0.55	(IV)
168985	Speciophylline	C_21_H_24_N_2_O_4_	368.43	5	1	1.62	No	0.55	C (IV)

Note: MF molecular formula, MW molecular weight (g/mol), HBA hydrogen bond acceptors, HBD hydrogen bond donors, BBB blood–brain barrier permeability, BA bioavailability score, LogP used is XLogP3, EP endpoint, C carcinogenicity, I immunotoxicity, Class IV (300 < LD_50_ ≤ 2000), Class V (2000 < LD_50_ ≤ 5000).

**Table 2:** Putative therapeutic target information of MS against AD.

Number	Gene ID	Gene Symbol	Protein Description
1	P31749	AKT1	RAC-alpha serine/threonine-protein kinase
2	P04406	GAPDH	Glyceraldehyde-3-phosphate dehydrogenase
3	P42574	CASP3	Caspase-3
4	P00533	EGFR	Epidermal growth factor receptor
5	P49841	GSK3B	Glycogen synthase kinase-3 beta
6	P40763	STAT3	Signal transducer and activator of transcription 3
7	P03372	ESR1	Estrogen receptor
8	P14780	MMP9	Matrix metalloproteinase-9
9	P19838	NFκB1	Nuclear factor NF-kappa-B p105 subunit
10	P42345	MTOR	Serine/threonine-protein kinase mTOR
11	P56817	BACE1	Beta-secretase 1
12	O00206	TLR4	Toll-like receptor 4
13	P42336	PIK3CA	Phosphatidylinositol 4
14	P49768	PSEN1	Presenilin-1
15	Q16236	NFE2L2	Nuclear factor erythroid 2-related factor 2
16	O14746	TERT	Telomerase reverse transcriptase
17	P27986	PIK3R1	Phosphatidylinositol 3-kinase regulatory subunit alpha
18	P04629	NTRK1	High affinity nerve growth factor receptor
19	P29474	NOS3	Nitric oxide synthase 3, endothelial
20	P10275	AR	Androgen receptor
21	P04035	HMGCR	3-hydroxy-3-methylglutaryl-coenzyme A reductase
22	Q92731	ESR2	Estrogen receptor beta
23	P06241	FYN	Tyrosine-protein kinase Fyn
24	P09874	PARP1	Poly [ADP-ribose] polymerase 1
25	Q00535	CDK5	Cyclin-dependent kinase 5
26	O14672	ADAM10	Disintegrin and metalloproteinase domain-containing protein 10
27	P78536	ADAM17	Disintegrin and metalloproteinase domain-containing protein 17
28	P05164	MPO	Myeloperoxidase
29	P22303	ACHE	Acetylcholinesterase
30	P11166	SLC2A1	Solute carrier family 2
31	P08473	MME	Neprilysin
32	P49810	PSEN2	Presenilin-2
33	P07339	CTSD	Cathepsin D
34	P07333	CSF1R	Macrophage colony-stimulating factor 1 receptor
35	P30556	AGTR1	Type-1 angiotensin II receptor
36	P07949	RET	Proto-oncogene tyrosine-protein kinase receptor Ret
37	P11511	CYP19A1	Aromatase
38	P21397	MAOA	Amine oxidase [flavin-containing] A
39	P06276	BCHE	Cholinesterase
40	Q5S007	LRRK2	Leucine-rich repeat serine/threonine-protein kinase 2
41	P29475	NOS1	Nitric oxide synthase 1
42	Q9UHD2	TBK1	Serine/threonine-protein kinase TBK1
43	P55210	CASP7	Caspase-7
44	Q14289	PTK2B	Protein-tyrosine kinase 2-beta
45	Q01959	SLC6A3	Sodium-dependent dopamine transporter
46	Q92542	NCSTN	Nicastrin
47	P09211	GSTP1	Glutathione S-transferase P
48	Q9HBA0	TRPV4	Transient receptor potential cation channel subfamily V member 4
49	P11217	PYGM	Glycogen phosphorylase
50	P06737	PYGL	Glycogen phosphorylase
51	P13569	CFTR	Cystic fibrosis transmembrane conductance regulator
52	Q8WW43	APH1B	Gamma-secretase subunit APH-1B
53	Q9UNY4	F2	Transcription termination factor 2
54	Q99720	SIGMAR1	Sigma non-opioid intracellular receptor 1
55	Q15078	CDK5R1	Cyclin-dependent kinase 5 activator 1
56	P17787	CHRNB2	Neuronal acetylcholine receptor subunit beta-2
57	P42892	ECE1	Endothelin-converting enzyme 1
58	P10253	GAA	Lysosomal alpha-glucosidase
59	P36544	CHRNA7	Neuronal acetylcholine receptor subunit alpha-7
60	Q99714	HSD17B10	3-hydroxyacyl-CoA dehydrogenase type-2

**Table 3:** Interaction parameters of core targets with the compounds of MS.

Target	Compound	Hydrogen Bonding	Alkyl	Pi-Alkyl	Pi-Sigma	Pi-Pi
AKT1	Acetylursolic acid	ASN-54 (5.37) ARG-273 (3.09) TYR-272 (4.59)	VAL-270 (4.28, 5.42) LYS-268 (4.44) LEU-264 (4.80, 5.88)	TRP-80 (4.09, 6.79) TYR-272 (6.22)	TRP-80 (3.25, 3.80)	-
	Beta-Sitosterol	SER-205 (4.00) ALA-212 (4.10)	VAL-270 (4.96, 5.33) LEU-264 (4.55, 5.29, 5.70) LEU-210 (4.12, 4.87)	TRP-80 (4.63, 5.53, 5.67, 5.82)	-	-
	Isomitraphylline	VAL-271 (4.59) SER-205 (3.66) LYS-268 (4.56)	LYS-268 (3.92, 5.29) LEU-264 (5.41)	TYR-263 (5.66) TRP-80 (4.35)	TRP-80 (4.35)	-
	Speciophylline	GLU-85 (3.61) VAL-83 (4.94) ARG-273 (5.05) TYR-326 (5.87)	ILE-84 (5.61) ARG-86 (4.27)	-	-	LEU-295 (8.96)
GSK3B	Acetylursolic acid	ASN-95 (4.58) LYS-292 (4.94)	ARG-96 (6.32) LYS-292 (4.12, 4.44, 5.21) PRO-294 (4.62, 7.01) VAL-263 (5.19) VAL-267 (5.91)	PHE-93 (4.36, 5.20) PHE-293 (4.38)	-	-
	Beta-Sitosterol	-	PRO-294 (4.58, 4.73) VAL-87 (5.10) LYS-292 (4.51, 6.40)	PHE-67 (4.52, 5.06) PHE-93 (5.22, 5.30)	PHE-93 (4.81)	-
	Isomitraphylline	ARG-96 (5.23) GLN-89 (4.03)	ARG-96 (5.00) LYS-292 (5.67) PRO-294 (4.12) VAL-267 (4.61)	PRO-294 (5.49) PHE-67 (4.13) PHE-293 (4.54, 5.25)	-	-
	Speciophylline	ASP-90 (3.41, 4.91) GLN-89 (3.65) LEU-88 (5.31) GLU-97 (5.33) ARG-96 (3.71)	ARG-96 (4.45, 5.49) LYS-292 (5.40)	PRO-294 (4.60) PHE-93 (3.71)	-	-
NFκB1	Acetylursolic acid	GLU-68 (4.60)	ALA-69 (5.36, 3.72) LEU-16 (6.02)	PHE-80 (6.71, 5.49) PHE-11 (5.48, 7.62)	-	-
	Beta-Sitosterol	ASP-16 (3.34)	ALA-69 (4.54, 3.72) LEU-15 (6.39)	PHE-11 (6.20) PHE-80 (6.50, 6.68) LEU-15 (5.88)	PHE-11 (4.98)	-
	Isomitraphylline	GLU-68 (4.66) LYS-85 (4.21)	ALA-69 (6.17) LYS-85 (4.02)	PHE-80 (6.90)	ALA-69 (4.11)	-
	Speciophylline	GLN-78 (3.58) LYS-85 (4.37, 4.75)	LYS-85 (3.70, 4.30)	-	ALA-69 (3.72)	PHE-11 (7.13)
BACE1	Acetylursolic acid	GLN-53 (4.58)	ARG-50 (4.12, 5.90) LEU-27 (5.41)	TYR-51 (6.35)	-	-
	Beta-Sitosterol	ASP-32 (4.33)	ILE-110 (3.84, 5.06)	TYR-71 (4.61, 5.61)	-	-
	Isomitraphylline	SER-46P (5.70)	MET-2 (3.35, 6.64)	PHE-47P (4.31)	ASN-92 (4.35)	-
	Speciophylline	THR-232 (4.18) PHE-108 (4.89)	ILE-110 (5.09, 5.85)	-	-	TYR-71 (5.06)

## Data Availability

All data generated or analyzed during this study are included in this published article and its Supplementary Information Files.

## Supplementary Materials

The following supporting information can be downloaded at https://www.mdpi.com/article/10.3390/ijms252313201/s1.

## Abbreviation

AChE	Acetylcholinesterase
AD	Alzheimer’s disease
ADME	Absorption, distribution, metabolism, and excretion
AE	Atropine equivalents
Aβ	Amyloid-beta
BBB	Blood–Brain barrier
BChE	Butyrylcholinesterase
BP	Biological processes
CC	Cellular components
DWE	Distill water extract
EE	Ethanolic extract
FDR	False discovery rate
GAE	Gallic acid equivalents
GHS	Globally harmonized system
GO	Gene ontology
HBA	Hydrogen bond acceptors
HBD	Hydrogen bond donors
KEGG	Kyoto Encyclopedia of Genes and Genomes
log2 FC	Log 2 Fold change
MDA	Malondialdehyde
MF	Molecular functions
MOE	Molecular operating environment
MS	*Mitragyna speciosa*
NGF	Nerve growth factor
NMDA	N-methyl-D-aspartic acid or N-methyl-D-aspartate
PDB	Protein Data Bank
PHWE	Pressurized hot water extract
QE	Quercetin equivalents
RNS	Reactive nitrogen species
ROS	Reactive oxygen species
SOD	Superoxide dismutase
UPS	Ubiquitin–proteasome system
