# Supplementary material for: Network Pharmacology, Molecular Docking, and In Vitro Insights into the Potential of Mitragyna speciosa for Alzheimer’s Disease
Source: Int J Mol Sci. 2024 Dec 8;25(23):13201. doi: 10.3390/ijms252313201 (PMC11642099; doi:10.3390/ijms252313201)
Supplement: Supplementary file 1 [file ijms-25-13201-s001.zip › Figure S1.The distribution of MS and AD overlapping genes.pdf]

Supplementary file (Figure S1)

A

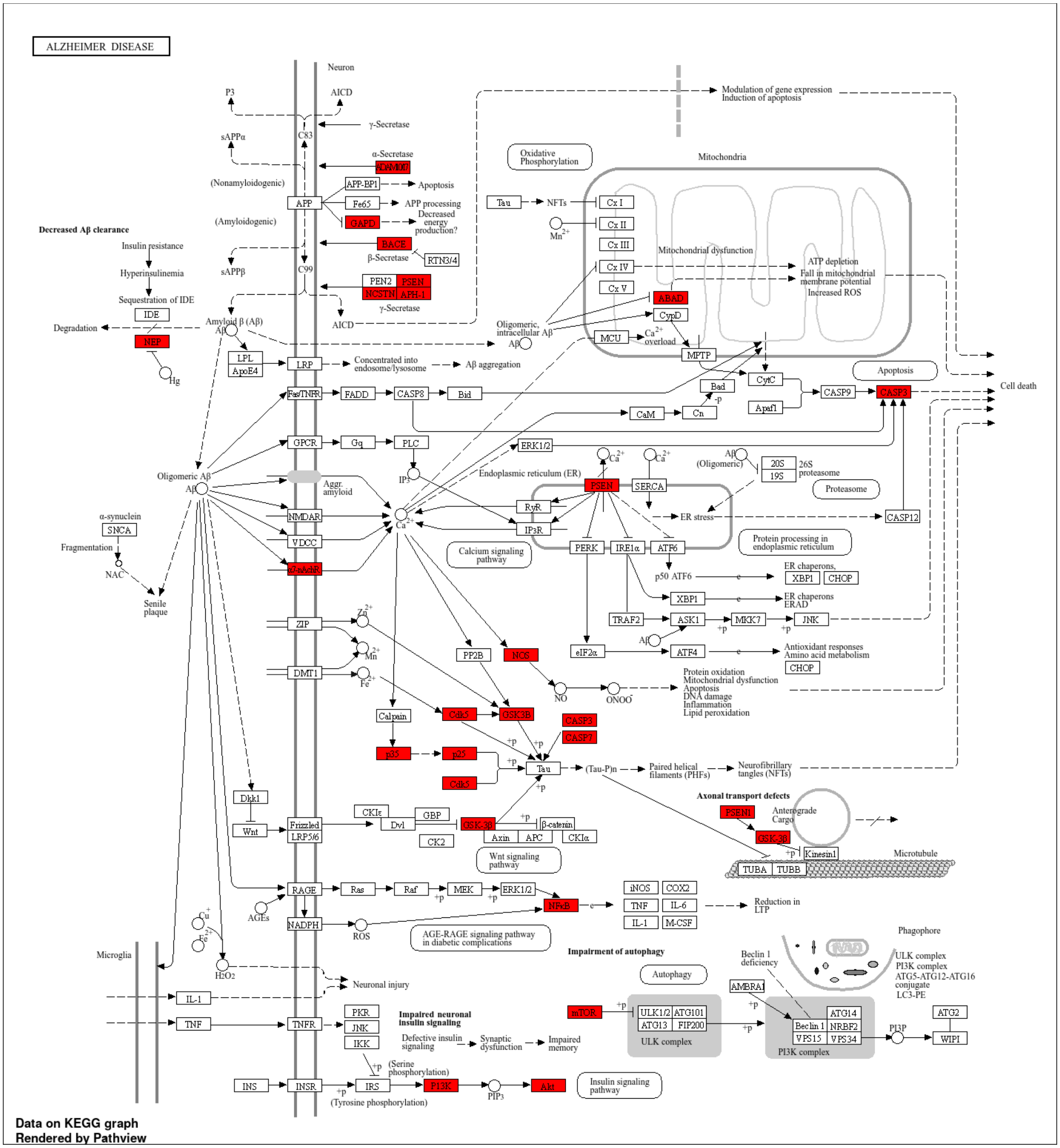

B

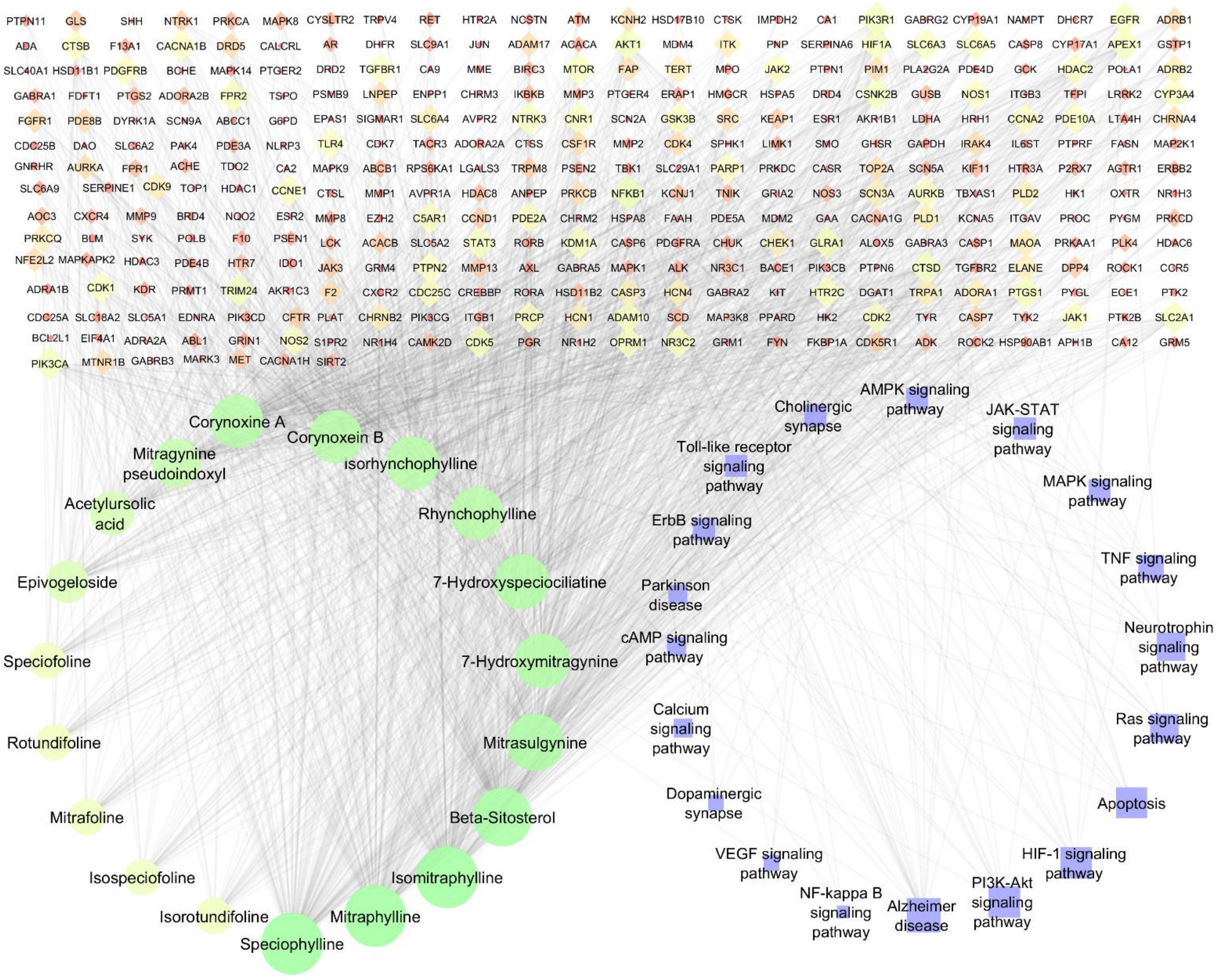

Figure S1. The distribution of MS and AD overlapping genes (A) KEGG pathway for Alzheimer's disease (hsa05010), the figure obtained under permission and guidelines [28] (red font represented the possible therapeutic target of MS involved in the AD pathway), (B) The "compound-gene targets-pathways" network (green ellipse, blue square, and orange diamond denote compounds, potential pathways, and gene targets, respectively).
